# Supplementary material for: Perceived usefulness of trauma audit filters in urban India: a mixed-methods multicentre Delphi study comparing filters from the WHO and low and middle-income countries
Source: BMJ Open. 2022 Jun 8;12(6):e059948. doi: 10.1136/bmjopen-2021-059948 (PMC9185581; doi:10.1136/bmjopen-2021-059948)
Supplement: Supplementary data [file bmjopen-2021-059948supp001.pdf]

Supplemental material

01/03/2022

Supplemental material

Analysis of the pre-Delphi by the core team

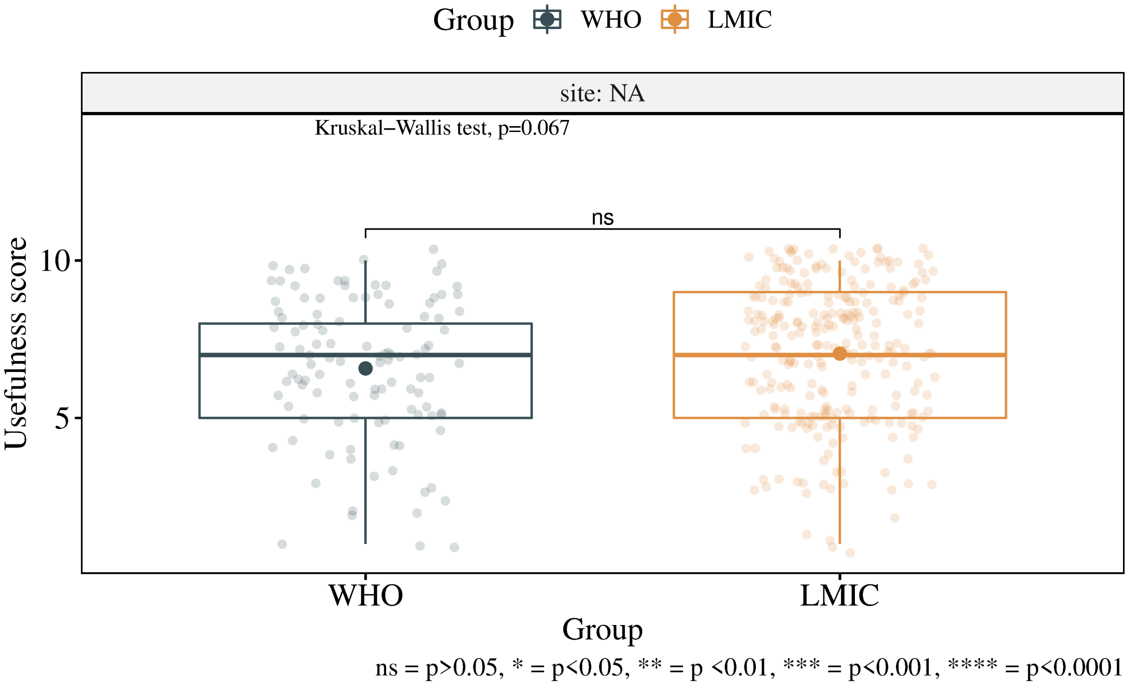

Figure 1: Pre-Delphi: Usefulness scores and level of significance for trauma audit filters by source group

All initial audit filters

Table 1: All initial audit filters. n = 67

Group	ID	Audit filter
**WHO**	1	Response time of surgeons
**WHO**	2	Availability of operating room
**WHO**	3	Sequential GCS in the emergency department of trauma patients with a diagnosis of skull fracture, intracranial injury or spinal cord injury
**WHO**	4	At least hourly, determination and recordings of blood pressure, pulse, respirations, temperature, Glasgow Coma Scale (GCS) score and intake and output (I&O) measurements for a major or severe trauma patient done. Beginning with arrival in the resuscitation area and including time spent in radiology up to admission to the operating room or ICU, death, or transfer to another hospital
**WHO**	5	Documentation of history and physical examination by a doctor
**WHO**	6	Head computerized tomography (CT) scan done within 2 hours of arrival at hospital for a non-transferred patient with Glasgow Coma Scale score <13
**WHO**	7	Endotracheal tube or surgical airway performed before leaving resuscitation area in patient with Glasgow Coma Scale score <8
**WHO**	8	No laparotomy within 1 hour of arrival at the hospital for patient with abdominal injuries and hypotension (systolic BP <90)
**WHO**	9	Laparotomy not performed (within 4 hours to or 24 hours after admission depending on hospital practice)
**WHO**	10	Craniotomy performed within 4 hours of arrival to the emergency department, for drainage of epidural or subdural haematoma
**WHO**	11	Abdominal, thoracic, vascular or cranial surgery after 24 hours
**WHO**	12	Unplanned return to operating theatre within 48 hours of initial procedure
**WHO**	13	Patient requiring re-intubation of the airway within 48 hours of extubation
**WHO**	14	Operative treatment of gunshot wound to the abdomen
**WHO**	15	Fixation of femoral fracture in adult patient within 24 hours of arrival to emergency department
**WHO**	16	All delays in identification of injuries
**WHO**	17	All trauma deaths
**WHO**	18	Required equipment, shared with other departments (e.g. fluid warmer, ventilator), not immediately available when requested
**WHO**	19	Non-compliance with institutional protocols
**WHO**	20	All major complications (e.g. deep venous thrombosis, pulmonary embolus, decubitus ulcers
**Ghana and Cameroon**	21	Vital signs recorded within 15 minutes of arrival (must include breathing assessment, heart rate, blood pressure, oxygen saturation if available)
**Ghana**	22	Senior medical officer made aware of patient with difficulty breathing, OR shock present at triage (HR >100, OR SBP <110)* OR oxygen saturation <95% within 5 minutes of initial assessment
**Ghana**	23	The clinician did assess airway patency by asking the patient a question and listening for a response
**Ghana**	24	Basic airway manoeuvre assistance (i.e. sweep, chin-lift-jaw-thrust, oral or nasal airway, suction) performed for patient with difficulty or obstructed breathing.
**Ghana**	25	Examination for pneumo- hemothorax done by listening to both sides of the chest with a stethoscope and bilateral percussion, within 60 minutes of patient arrival to emergency department

Group	ID	Audit filter
**Ghana**	26	Chest tube placed within 30 min of patient arrival in patient with suspected or confirmed pneumo- or hemothorax and oxygen saturation less than 98%
**Ghana**	27	Large bore IV was placed within 15 min of patient arrival to the emergency department
**Ghana and Cameroon**	28	Pressure applied to external bleeding at patient arrival, and maintained until definitive control is performed
**Ghana and Cameroon**	29	If AVPU is not ‘A’ AND the patient is not in shock, the head of the bed is elevated to 45 degrees.
**Ghana**	30	Reduction and/or splinting with analgesia made for a long bone fracture within 2 h of admission or prior to transfer
**Ghana and Cameroon**	31	Patient is completely undressed, fully examined and covered for privacy within 30 min of arrival
**Ghana**	32	Fluid order for a burn patient not done using the Parkland formula is recorded within 1 h for burns over 15% total body surface area that occurred less than 24 h from patient arrival
**Cameroon**	33	No sufficient history from patient or next best historian obtained.
**Cameroon**	34	No emergency department team prepared and ready with sufficient staff before patient arrival.
**Cameroon**	35	Burn patient did not receive 2-4 mL of crystalloid solution per kilogram body weight per percent body surface burn within 24 h of injury
**Cameroon**	36	Senior attending physician alerted when airway is compromised, usage jaw thrust, chin lift, ORA/NPA, or suction to open airway
**Cameroon**	37	Assessment of mouth/throat for foreign bodies and debris made in patient that has difficulty breathing, within 10 minutes of arrival to emergency department
**Cameroon**	38	Breathing assessment made within 15 min of arrival to emergency department
**Cameroon**	39	Establish two large bore IVs within 15 min of arrival.
**Cameroon**	40	Patient assessed for hypovolemia when presenting with hypotension and tachycardia or suspected intra-abdominal bleeding, femoral shaft fracture, or pelvic fracture.
**Cameroon**	41	Neurological assessment made with AVPU.
**Cameroon**	42	If patient arrived on spinal board, when appropriate patient is subsequently safely logrolled off board.
**Cameroon**	43	Open wounds are treated and covered
**Cameroon**	44	Chest X-ray performed for pneumothorax, hemothorax, and flail chest diagnosis.
**Cameroon**	45	Laparotomy not done within 1 h of arrival to hospital in a patient with abdominal injuries and systolic blood pressure <90
**Cameroon**	46	Immobilization and imaging performed in a patient with suspected spine injury, within 4 hours of arrival to the emergency department
**Cameroon**	47	I.v antibiotics given within 1 hour of arrival to the emergency department in a patient with an open fracture
**Cameroon**	48	Long bone fracture is reduced with analgesia and/or splinted within 4 h of admission or prior to transfer.
**Cameroon**	49	Operation for irrigation and debridement within 12 h from arrival to emergency department for an open fracture
**Cameroon**	50	Before patient arrival, emergency department is adequately stocked with essential supplies.
**Cameroon**	51	Referral made within 15 min of determination if treatment of injury is beyond site’s capabilities (i.e. not able to operate).
**Cameroon**	52	Second surgery within 48 h of previous operation

Berg J, *et al. BMJ Open* 2022; 12:e059948. doi: 10.1136/bmjopen-2021-059948

Group	ID	Audit filter
**Cameroon**	53	Unplanned return to operating theater.
**Cameroon**	54	Unplanned intubation.
**Cameroon**	55	Unplanned return to intensive care unit.
**Cameroon**	56	Unplanned readmission.
**Cameroon**	57	No ventilation done with bag-valve mask in patient who can not self ventilate.
**Cameroon**	58	Intubation performed in patient with a GCS score of 8 or less.
**Cameroon**	59	Thoracostomy within 30 min of arrival to emergency department in patient with pneumothorax or haemothorax
**Cameroon**	60	Patient body temperature is maintained by giving warmed IV fluids and blood to prevent hypothermia
**Cameroon**	61	If intracranial pressure <20 mm Hg, patient treated with mannitol or hypertonic saline
**Cameroon**	62	Operation for sub or epidural hematoma within 3 hours of arrival to emergency department
**Cameroon**	63	No FAST exam performed to evaluate for pericardial effusion.
**Cameroon**	64	Pericardiocentesis performed immediately in patient with detected pericardial effusion and significant hypotension.
**Cameroon**	65	FAST exam performed within 30 minutes from arrival to the emegency department to exclude hemoperitoneum.
**Cameroon**	66	If GCS <13 at initial assessment or if GCS <15 2 h after injury, then skull X-ray is given.
**Cameroon**	67	Patient arrived with spine immobilized on spinal board or rigid object of similar function

Berg J, *et al. BMJ Open* 2022; 12:e059948. doi: 10.1136/bmjopen-2021-059948

Welcome to the first round of the Trauma Audit Filters Trial delphi survey to develop locally relevant audit filters!

Your task is to rate each statement on the following pages on a scale from 1 to 10, where 1 represents not useful and 10 very useful. A statement is useful if:

1. Failure to perform the action depicted in the statement is likely to result in poor patient outcome or complications 2. It is feasible to collect data on the statement in your setting.

If you want, you may comment on statements. For example, if you find that the time frame of a statement is not feasible, you may write so in the comment, and suggest a new time frame. At the end of the survey you may suggest new statements, or filters, that you think would be useful to track quality of trauma care at your centre.

Thank you for participating!

Please send an email to Martin Gerdin Wärnberg at martin.gerdin@ki.se if you have any questions.

Section A: Audit filters

A1. **Filter ID: TAFT3Sequential GCS in the emergency department of trauma patients with a diagnosis of skull fracture, intracranial injury or spinal cord injury**

	1 - Not useful	2	3	4	5	6	7	8	9	10 - Very useful
Rate the usefulness of this audit filter	☐	☐	☐	☐	☐	☐	☐	☐	☐	☐

A2. **Add a comment about this audit filter**

A3. **Filter ID: TAFT5Documentation of history and physical examination by a doctor**

	1 - Not useful	2	3	4	5	6	7	8	9	10 - Very useful
Rate the usefulness of this audit filter	☐	☐	☐	☐	☐	☐	☐	☐	☐	☐

A4. **Add a comment about this audit filter**

A5. **Filter ID: TAFT6Head computerized tomography (CT) scan done within 2 hours of arrival at hospital for a non-transferred patient with Glasgow Coma Scale score 90**

	1 - Not useful	2	3	4	5	6	7	8	9	10 - Very useful
Rate the usefulness of this audit filter	☐	☐	☐	☐	☐	☐	☐	☐	☐	☐

A6. **Add a comment about this audit filter**

A7. **Filter ID: TAFT14Operative treatment of gunshot wound to the abdomen**

	1 - Not useful	2	3	4	5	6	7	8	9	10 - Very useful
Rate the usefulness of this audit filter	☐	☐	☐	☐	☐	☐	☐	☐	☐	☐

A8. **Add a comment about this audit filter**

Section B: Audit filters

B1. **Filter ID: TAFT15Fixation of femoral fracture in adult patient within 24 hours of arrival to emergency department**

	1 - Not useful	2	3	4	5	6	7	8	9	10 - Very useful
Rate the usefulness of this audit filter	☐	☐	☐	☐	☐	☐	☐	☐	☐	☐

B2. **Add a comment about this audit filter**

B3. **Filter ID: TAFT21Vital signs recorded within 15 minutes of arrival to emergency department (must include breathing assessment, heart rate, blood pressure, oxygen saturation if available)**

	1 - Not useful	2	3	4	5	6	7	8	9	10 - Very useful
Rate the usefulness of this audit filter	☐	☐	☐	☐	☐	☐	☐	☐	☐	☐

B4. **Add a comment about this audit filter**

B5. **Filter ID: TAFT22Senior medical officer made aware of patient with difficulty breathing, or shock present at triage (HR >100, OR SBP**

	1 - Not useful	2	3	4	5	6	7	8	9	10 - Very useful
Rate the usefulness of this audit filter	☐	☐	☐	☐	☐	☐	☐	☐	☐	☐

B6. **Add a comment about this audit filter**

B7. **Filter ID: TAFT23The clinician did assess airway patency by asking the patient a question and listening for a response**

	1 - Not useful	2	3	4	5	6	7	8	9	10 - Very useful
Rate the usefulness of this audit filter	☐	☐	☐	☐	☐	☐	☐	☐	☐	☐

B8. **Add a comment about this audit filter**

Section C: Audit filters

C1. **Filter ID: TAFT24Basic airway manoeuvre assistance (i.e. sweep, chin-lift-jaw-thrust, oral or nasal airway, suction) performed for patient with difficulty or obstructed breathing.**

	1 - Not useful	2	3	4	5	6	7	8	9	10 - Very useful
Rate the usefulness of this audit filter	☐	☐	☐	☐	☐	☐	☐	☐	☐	☐

C2. **Add a comment about this audit filter**

C3. **Filter ID: TAFT25Examination for pneumo- hemothorax done by listening to both sides of the chest with a stethoscope within 15 minutes of patient arrival to emergency department**

	1 - Not useful	2	3	4	5	6	7	8	9	10 - Very useful
Rate the usefulness of this audit filter	☐	☐	☐	☐	☐	☐	☐	☐	☐	☐

C4. **Add a comment about this audit filter**

C5. **Filter ID: TAFT26Chest tube placed within 30 min of patient arrival in patient with suspected or confirmed pneumo- or hemothorax and oxygen saturation less than 98%**

	1 - Not useful	2	3	4	5	6	7	8	9	10 - Very useful
Rate the usefulness of this audit filter	☐	☐	☐	☐	☐	☐	☐	☐	☐	☐

C6. **Add a comment about this audit filter**

C7. **Filter ID: TAFT27Large bore IV was placed within 15 min of patient arrival to the emergency department**

Rate the usefulness of this audit filter: 1 - Not useful ☐ 2 ☐ 3 ☐ 4 ☐ 5 ☐ 6 ☐ 7 ☐ 8 ☐ 9 ☐ 10 - Very useful ☐

C8. **Add a comment about this audit filter**

Section D: Audit filters

D1. **Filter ID: TAFT28Pressure applied to external bleeding at patient arrival to the emergency department, and maintained until definitive control is performed**

Rate the usefulness of this audit filter: 1 - Not useful ☐ 2 ☐ 3 ☐ 4 ☐ 5 ☐ 6 ☐ 7 ☐ 8 ☐ 9 ☐ 10 - Very useful ☐

D2. **Add a comment about this audit filter**

D3. **Filter ID: TAFT30Reduction and/or splinting with analgesia made for a long bone fracture within 2 hours of admission or prior to transfer**

Rate the usefulness of this audit filter: 1 - Not useful ☐ 2 ☐ 3 ☐ 4 ☐ 5 ☐ 6 ☐ 7 ☐ 8 ☐ 9 ☐ 10 - Very useful ☐

D4. **Add a comment about this audit filter**

D5. **Filter ID: TAFT35Burn patient did receive 2-4 mL of crystalloid solution per kilogram body weight per percent body surface burn within 24 hours of injury**

	1 - Not useful	2	3	4	5	6	7	8	9	10 - Very useful
Rate the usefulness of this audit filter	☐	☐	☐	☐	☐	☐	☐	☐	☐	☐

D6. **Add a comment about this audit filter**

D7. **Filter ID: TAFT36Senior attending physician alerted when airway is compromised, usage jaw thrust, chin lift, ORA/NPA, or suction to open airway**

	1 - Not useful	2	3	4	5	6	7	8	9	10 - Very useful
Rate the usefulness of this audit filter	☐	☐	☐	☐	☐	☐	☐	☐	☐	☐

D8. **Add a comment about this audit filter**

Section E: Audit filters

E1. **Filter ID: TAFT37Assessment of mouth/throat for foreign bodies and debris made in patient that has difficulty breathing, within 10 minutes of arrival to emergency department**

	1 - Not useful	2	3	4	5	6	7	8	9	10 - Very useful
Rate the usefulness of this audit filter	☐	☐	☐	☐	☐	☐	☐	☐	☐	☐

E2. **Add a comment about this audit filter**

E3. **Filter ID: TAFT38Breathing assessment made within 15 min of arrival to emergency department**

	1 - Not useful	2	3	4	5	6	7	8	9	10 - Very useful
Rate the usefulness of this audit filter	☐	☐	☐	☐	☐	☐	☐	☐	☐	☐

E4. **Add a comment about this audit filter**

E5. **Filter ID: TAFT40Patient assessed for hypovolemia when presenting with hypotension and tachycardia or suspected intra-abdominal bleeding, femoral shaft fracture, or pelvic fracture.**

	1 - Not useful	2	3	4	5	6	7	8	9	10 - Very useful
Rate the usefulness of this audit filter	☐	☐	☐	☐	☐	☐	☐	☐	☐	☐

E6. **Add a comment about this audit filter**

E7. **Filter ID: TAFT45Laparotomy done within 1 hour of arrival to the emergency department in a patient with abdominal injuries and systolic blood pressure**

	1 - Not useful	2	3	4	5	6	7	8	9	10 - Very useful
Rate the usefulness of this audit filter	☐	☐	☐	☐	☐	☐	☐	☐	☐	☐

E8. **Add a comment about this audit filter**

Section F: Audit filters

F1. **Filter ID: TAFT46Immobilization and imaging performed in a patient with suspected spine injury, within 4 hours of arrival to the emergency department**

Rate the usefulness of this audit filter: 1 - Not useful ☐ 2 ☐ 3 ☐ 4 ☐ 5 ☐ 6 ☐ 7 ☐ 8 ☐ 9 ☐ 10 - Very useful ☐

F2. **Add a comment about this audit filter**

F3. **Filter ID: TAFT47I.v antibiotics given within 1 hour of arrival to the emergency department in a patient with an open fracture**

Rate the usefulness of this audit filter: 1 - Not useful ☐ 2 ☐ 3 ☐ 4 ☐ 5 ☐ 6 ☐ 7 ☐ 8 ☐ 9 ☐ 10 - Very useful ☐

F4. **Add a comment about this audit filter**

F5. **Filter ID: TAFT49Operation for irrigation and debridement within 12 hours from arrival to emergency department for an open fracture**

Rate the usefulness of this audit filter: 1 - Not useful ☐ 2 ☐ 3 ☐ 4 ☐ 5 ☐ 6 ☐ 7 ☐ 8 ☐ 9 ☐ 10 - Very useful ☐

F6. **Add a comment about this audit filter**

F7. **Filter ID: TAFT58Intubation performed in patient with a GCS score of 8 or less within 30 minutes of arrival to emergency department.**

Rate the usefulness of this audit filter: 1 - Not useful ☐ 2 ☐ 3 ☐ 4 ☐ 5 ☐ 6 ☐ 7 ☐ 8 ☐ 9 ☐ 10 - Very useful ☐

F8. **Add a comment about this audit filter**

Section G: Audit filters

G1. **Filter ID: TAFT62Operation for sub or epidural hematoma within 3 hours of arrival to emergency department**

Rate the usefulness of this audit filter: 1 - Not useful ☐ 2 ☐ 3 ☐ 4 ☐ 5 ☐ 6 ☐ 7 ☐ 8 ☐ 9 ☐ 10 - Very useful ☐

G2. **Add a comment about this audit filter**

G3. **Filter ID: TAFT65FAST exam performed within 30 minutes from arrival to the emegency department to exclude hemoperitoneum.**

Rate the usefulness of this audit filter: 1 - Not useful ☐ 2 ☐ 3 ☐ 4 ☐ 5 ☐ 6 ☐ 7 ☐ 8 ☐ 9 ☐ 10 - Very useful ☐

G4. **Add a comment about this audit filter**

G5. **If you wish to propose new audit filters, please enter them here.**
Separate your suggested filters with a semicolon, for example
Response time of surgeons; Neurological assessment made with AVPU;
