# Supplementary material for: Perceived usefulness of trauma audit filters in urban India: a mixed-methods multicentre Delphi study comparing filters from the WHO and low and middle-income countries
Source: BMJ Open. 2022 Jun 8;12(6):e059948. doi: 10.1136/bmjopen-2021-059948 (PMC9185581; doi:10.1136/bmjopen-2021-059948)
Supplement: Author's manuscript [file bmjopen-2021-059948.draft_revisions.pdf]

BMJ Open is committed to open peer review. As part of this commitment we make the peer review history of every article we publish publicly available.

When an article is published we post the peer reviewers' comments and the authors' responses online. We also post the versions of the paper that were used during peer review. These are the versions that the peer review comments apply to.

The versions of the paper that follow are the versions that were submitted during the peer review process. They are not the versions of record or the final published versions. They should not be cited or distributed as the published version of this manuscript.

BMJ Open is an open access journal and the full, final, typeset and author-corrected version of record of the manuscript is available on our site with no access controls, subscription charges or pay-per-view fees (http://bmjopen.bmj.com).

If you have any questions on BMJ Open's open peer review process please email info.bmjopen@bmj.com

BMJ Open

Perceived usefulness of trauma audit filters in urban India: a multicenter Delphi study comparing filters from the World Health Organization and Low-and-Middle-Income Countries

Journal:	*BMJ Open*
Manuscript ID	bmjopen-2021-059948
Article Type:	Original research
Date Submitted by the Author:	10-Dec-2021
Complete List of Authors:	Berg, Johanna; Karolinska Institutet, Department of Global Public Health; Skånes universitetssjukhus Malmö, Department of Emergency and Internal medicine<br>Alvesson, Helle; Karolinska Institutet, Department of Global Public Health<br>Roy, Nobhojit; The George Institute for Global Health India; Karolinska Institutet, Department of Global Public Health<br>Ekelund, U; Lund University Faculty of Medicine, Department of Clinical Sciences Lund, Emergency Medicine<br>Bains, Lovenish; Maulana Azad Medical College, Department of Surgery<br>Chatterjee, Shamita; Seth Sukhlal Karnani Memorial Hospital, Department of Surgery<br>Bhattacharjee, Prosanta; Seth Sukhlal Karnani Memorial Hospital, Department of Surgery; RG Kar Medical College, Department of Surgery<br>Siddarth, David; Karolinska Institutet, Department of Global Public Health; Doctors for you<br>Gupta, Swati; Maulana Azad Medical College, Department of Radiodiagnosis and Imaging<br>Kamble, Jyoti; Tata Institute of Social Sciences; Doctors for you<br>Khajanchi, Monty; Seth GS Medical College and KEM Hospital, Department of Surgery; WHO Collaborating Centre for research on surgical care delivery in low and middle income countries<br>Lal, Pawanindra; Maulana Azad Medical College, Department of Surgery<br>Malhotra, Vikas; Maulana Azad Medical College, Department of ENT and Head & Neck Surgery<br>Meher, Ravi; Maulana Azad Medical College, Department of ENT and Head & Neck Surgery<br>Mishra, Anurag; Maulana Azad Medical College, Department of Surgery<br>Mohan, L N; Vydehi Institute of Medical Sciences and Research Centre, Surgery<br>Petzold, Max; University of Gothenburg Sahlgrenska Academy, Public Health and Community Medicine<br>Saxena, Ritu; Lok Nayak Hospital, Department of Accident & Emergency<br>Shrivastava, Prabhat; Maulana Azad Medical College, Department of Burns and Plastic Surgery<br>Singh, Rajdeep; Maulana Azad Medical College, Department of Surgery<br>Soni, Kapil; All India Institute of Medical Sciences, Department of Critical and Intensive Care<br>Sural, Sumit; Maulana Azad Medical College, Department of Orthopaedic

	Surgery<br>Gerdin Wärnberg, Martin ; Karolinska Institutet, Department of Global Public Health; Karolinska University Hospital, Function Perioperative Medicine and Intensive Care

Keywords:	ACCIDENT & EMERGENCY MEDICINE, TRAUMA MANAGEMENT, Trauma management < ORTHOPAEDIC & TRAUMA SURGERY

I, the Submitting Author has the right to grant and does grant on behalf of all authors of the Work (as defined in the below author licence), an exclusive licence and/or a non-exclusive licence for contributions from authors who are: i) UK Crown employees; ii) where BMJ has agreed a CC-BY licence shall apply, and/or iii) in accordance with the terms applicable for US Federal Government officers or employees acting as part of their official duties; on a worldwide, perpetual, irrevocable, royalty-free basis to BMJ Publishing Group Ltd ("BMJ") its licensees and where the relevant Journal is co-owned by BMJ to the co-owners of the Journal, to publish the Work in this journal and any other BMJ products and to exploit all rights, as set out in our licence.

The Submitting Author accepts and understands that any supply made under these terms is made by BMJ to the Submitting Author unless you are acting as an employee on behalf of your employer or a postgraduate student of an affiliated institution which is paying any applicable article publishing charge ("APC") for Open Access articles. Where the Submitting Author wishes to make the Work available on an Open Access basis (and intends to pay the relevant APC), the terms of reuse of such Open Access shall be governed by a Creative Commons licence – details of these licences and which Creative Commons licence will apply to this Work are set out in our licence referred to above.

Other than as permitted in any relevant BMJ Author's Self Archiving Policies, I confirm this Work has not been accepted for publication elsewhere, is not being considered for publication elsewhere and does not duplicate material already published. I confirm all authors consent to publication of this Work and authorise the granting of this licence.

For peer review only

Perceived usefulness of trauma audit filters in urban India: a multicenter Delphi study comparing filters from the World Health Organization and Low-and-Middle-Income Countries

Date: 10 December 2021

Title page

Word count: 3547

Corresponding author

Johanna Berg (JB)
Karolinska Institutet, Department of Global Public Health; Skåne University hospital, Emergency medicine, department of Emergency and Internal medicine.

e-mail: johanna.berg@ki.se

Postal address: Skånes Universitetssjukhus Malmö, Ruth Lundskogs Gata 5, 214 28, Malmö, SWEDEN

Authors and affiliations

Johanna Berg (JB), MD. Karolinska Institutet, Department of Global Public Health; Skåne University hospital, Emergency medicine, department of Emergency and Internal medicine.

Helle Mölsted Alvesson (HMA), PhD. Karolinska Institutet, Department of Global Public Health; Karolinska Institutet

Nobhojit Roy (NR) MD, MPH, PhD 1.The George Institute for Global Health, New Delhi, INDIA 2. School of Public Health & Preventive Medicine, Monash University, Melbourne, Vic, Australia 3. GPH Karolinska

Ulf Ekelund (UE), MD, PhD. Department of Clinical Sciences Lund, Emergency Medicine, Faculty of Medicine, Lund University, Lund, Sweden

Lovenish Bains (LB), MS Surgery, FNB, FACS, FIAGES, FAIS. Department of Surgery, Maulana Azad Medical College & associated Lok Nayak Hospital, New Delhi, India

Shamita Chatterjee (SC), MS, FMAS, FAIS. Professor, Department of Surgery, IPGME&R - Seth Sukhlal Karnani Memorial Hospital, Kolkata, India.

For peer review only

Prosanta Kumar Bhattacharjee (PKB), MS (Surgery), FMAS. Professor of Surgery, R G Kar Medical College, Kolkata. Formally Professor of Surgery, I P G M E & R / S S K M Hospital, Kolkata, India.

Siddarth David (SD), MSc, Disaster Management, Project Manager, Doctors For You, India and Department of Global Public Health, Karolinska Institute, Sweden.

Swati Gupta (SG), MD Radiology, DNB radiology, FRCR, Department of Radiodiagnosis and Imaging, Maulana Azad Medical College & associated Lok Nayak Hospital, New Delhi, India.

Jyoti Kamble (JK), Sr. Research Officer, M.Sc (Biochemistry), Doctors For You, Mumbai, and Department of Public Health, Tata Institute of Social Sciences, Mumbai, India.

Monty Khajanchi (MK), DNB Surgery, Assistant Professor Surgery at Seth GSMC and KEM Hospital, Mumbai. Consultant WHO Collaboration Centre for Research in Surgical Delivery in Low and Middle Income Countries

Pawanindra Lal (PL), MS, DNB, FRCS(Ed,Eng,Glasg, Irel), FACS,FAMS. Director Professor, Department of Surgery, Maulana Azad Medical College & Associated Lok Nayak Hospital,New Delhi, India.

Vikas Malhotra (VM), MS ENT, DNB ENT, FRCS(Glasg), MBA, Department of ENT and Head & Neck Surgery, Maulana Azad Medical College & associated Lok Nayak Hospital, New Delhi, India.

Ravi Meher (RM), MS ENT, DNB ENT, FACS, MNAMS, Department of ENT and Head & Neck Surgery, Maulana Azad Medical College & associated Lok Nayak Hospital, New Delhi, India.

Anurag Mishra (AM), MS, DNB, MNAMS, FAIS, FACS. Department of Surgery, Maulana Azad Medical College & associated Lok Nayak Hospital, New Delhi, India.

L N Mohan (LNM), MBBS, MS, FRCS. Professor of Surgery, Vydehi Institute of Medical Sciences and Research Centre, Bangalore, India

Max Petzold (MP), School of Public Health and Community Medicine, Institute of Medicine, University of Gothenburg, Gothenburg, Sweden

Ritu Saxena (RS), MBBS, MBA (DM), AFIH, Dy. Medical Superintendent, Department of Accident & Emergency, Lok Nayak Hospital, New Delhi, India

Prabhat Shrivastava (PS), MBBS, MS (Surgery), MCh (Plastic Surgery), FICS, FAIS, Consultant and Head, Department of Burns and Plastic Surgery, Lok Nayak Hospital and associated Maulana Azad Medical College, New Delhi, India.

Rajdeep Singh (RS), MS (Surgery), DNB, FRCS (Glasg), FACS. Department of Surgery, Maulana Azad Medical College & associated Lok Nayak Hospital, New Delhi, India.

Kapil Dev Soni (KDS),MD, Critical and Intensive Care,JPN Apex Trauma Center, All India Institute of Medical Sciences, New Delhi, India

For peer review only

Sumit Sural (SS), MS(Ortho.).Department of Orthopaedic Surgery, Maulana Azad Medical College & associated Lok Nayak Hospital, New Delhi, India

Martin Gerdin Wärnberg (MGW), MD, PhD. Karolinska Institutet, Department of Global Public Health; Karolinska University Hospital, Function Perioperative Medicine and Intensive Care

For peer review only

Abstract

Objective: To compare experts' perceived usefulness of audit filters from different sources, generate context-appropriate audit filters for trauma care in urban India and explore characteristics of audit filters that correlate to perceived usefulness.
Design: A mixed methods approach using a multi-center online Delphi technique.
Setting: Two large tertiary hospitals in urban India**.**
Methods: Filters were rated on a scale from 1-10 in terms of perceived usefulness, with the option to add new filters and comments. The filters were categorized into three groups depending on their origin (LMIC, WHO, New) and their scores compared. Significance was determined using Kruskal–Wallis followed by Wilcox-rank-sum tests. We performed a content analysis of the comments.
Results: 26 predefined and 15 new filter suggestions were evaluated. The filters had high usefulness scores (mean overall score 9.01 of 10), with the LMIC filters having significantly higher scores compared to those from WHO and those newly added. Three themes were identified in the content analysis relating to *medical relevance*, *feasibility* and *specificity*.
Conclusions: Audit filters from other LMIC were deemed highly useful in this context. This may indicate that the transferability of defined trauma audit filters between similar contexts is high and that these can provide a starting point when implemented as part of TQIP in low-resource settings.

Keywords: trauma care, trauma, injury, trauma quality improvement, trauma system, audit filter

For peer review only

Article summary

Strengths and limitations of this study

- This study is the first to evaluate the usefulness of trauma audit filters from different sources in a low-resource context.
- We used two separate study sites to perform parallel independent online Delphi surveys to compare the usefulness of audit filters for trauma care.
- Both sites scored and selected filters with the intent to implement them.
- The content analysis gave a deeper understanding of the characteristics of the audit filters.
- Due to the nature of the Delphi methodology, the statistical analysis is based on a convenience sample.

For peer review only

Introduction

Trauma, defined as the body's response to physical injury, accounts for 4,4 million deaths globally every year.[1] With nearly 90% of these occurring in Low-and-Middle Income Countries (LMIC), it has been estimated that over 2 million lives each year could be saved if fatality rates were reduced to the levels of High-Income Countries (HIC).[2] Strengthening trauma care is essential to the global health agenda. There is evidence that implementing trauma quality improvement programs decreases mortality and improves trauma care in both HIC and LMIC.[3–5] These programs are diverse and involve different approaches to improving care, one of which is audit filters.

Audit filters, in some cases also referred to as quality indicators, are predefined statements that define deviations from standard care. For example, an audit filter could state, *"Patient with a GCS < 8 is intubated within 2 hours"*. A case violating this filter would indicate sub-optimal care and the case flagged for review. High-quality evidence on the effects on outcomes after implementing trauma audit filters is lacking.[6] However, the use of audit filters is one technique promoted in the World Health Organization (WHO) guidelines for trauma quality improvement programs.[7] Their implementation requires more extensive resources since they are data-driven.[7]

Audit filters need to be modified to the context in which they are to be applied.[7,8] To generate context-relevant audit filters, previous studies have used the Delphi technique to identify appropriate filters from a group of experts.[9,10] There is limited experience evaluating the filters proposed by the WHO and their perceived usefulness in a low-resource setting.

India accounts for 20-25% of trauma mortality globally.[1] In-hospital mortality remains high, with one study reporting 30-day mortality of 21%, which is twice as high compared to data from registries in HIC.[11] More than 50% of these deaths have been reported to be preventable.[12] Identifying evidence-based strategies to improve in-hospital trauma care in India is vital.

With this background, this study aims to compare experts' perceived usefulness of audit filters from different sources, generate context-appropriate audit filters for trauma care in urban India and explore characteristics of audit filters that correlate to perceived usefulness.

Material and Methods

Patient and public involvement

We did not have any patient or public involvement in this study.

Design

This study was nested in the Trauma Audit Filter Trial (TAFT) (ClinicalTrials.gov, ID NCT03235388), which investigates the effect of audit filter implementation on mortality

For peer review only

and morbidity in adult trauma patients in urban India. We used the Delphi technique to facilitate consensus within a panel of experts. [13] The Delphi technique is based on structured, iterative, anonymous surveys where participants can rate and comment on statements. Between each iteration, researchers may report feedback to the panellists to facilitate the discussion. Definition of when consensus is reached in a Delphi is predefined but the exact methods used to define this vary between studies. The Delphi technique has been used in multiple areas of health care research, including selecting trauma audit filters.[9,10] It is considered particularly useful when investigating complex multidisciplinary problems in areas with limited previous research where considerable uncertainties still exist.

Setting

We conducted the study at two public teaching hospitals located in cities with a population of more than 15 million. Seth Sukhlal Karnani Memorial Hospital (SSKM) in Kolkata and Maulana Azad Medical College (MAMC) with associated Lok Nayak Hospital in Delhi. Both are public tertiary teaching hospitals that serve as referral hospitals. The study was conducted online between December 2018 and March 2019.

Ethical approvals

Ethical approvals exist as a part of the TAFT project. In Sweden from the Swedish Ethical Review Authority, 2017/930-31/2 dated 2017-06-07. At MAMC - F.1/IEC/MAMC/(57/02/2017/No113) dated 2017-07-19 and SSKM - IPGME&R/IEC/2017/396 dated 2017-08-21.

Identification of audit filters

We identified trauma audit filters from Cameroon[9], Ghana[10] and those proposed in the WHO guidelines.[7] These were selected based on their intended use in LMIC. The TAFT core research team, based in India, conducted an internal online Delphi survey to remove duplicate filters, prioritize filters deemed potentially appropriate for the urban setting in India and evaluate the online tool used for this trial. The panel consisted of five participants, four surgeons and one critical care/anaesthesia physician. All at the Professor or Associate professor level with extensive experience of clinical trauma work and research in urban India.

Sixty-seven audit filters were identified from three different sources, 47 from Cameroon[9] and Ghana[10] and 20 from WHO guidelines.[7] The online Delphi for the TAFT core team in India was performed in November 2018. The survey consisted of three rounds and response rates were 100% at all rounds. This resulted in 26 selected filters, 5 from WHO and 21 from LMIC sources. (Supplemental material: Table 1)

Participants

We purposefully sampled physicians, surgeons, nurses, and administrators with >5 years of professional experience and involvement in trauma care at the respective hospitals. This was facilitated through the ongoing TAFT project, where local principal investigators with

For peer review only - http://bmjopen.bmj.com/site/about/guidelines.xhtml

intimate knowledge of the sites could identify experts for inclusion. We chose this approach since considerable knowledge about the local conditions and organization, in addition to work experience, would be necessary to evaluate the usefulness of the filters at each site. Before executing the online surveys, a two-day meeting was arranged at the participating hospitals discussing trauma quality improvement programs and introducing the concept of audit filters to the participants.

A total of 27 individuals were invited; of these 25 agreed to participate. Participation remained high at both sites throughout rounds (mean participation/round 84%). The distribution of the professions differed across sites, with one site being represented by surgeons and physicians only. (Table: 1).

Table 1: Characteristics of participants

Characteristic	Site 1, N = 10[1]	Site 2, N = 15[1]
**Profession**		
Administrator	1 (10%)	0 (0%)
Nurse	2 (20%)	0 (0%)
Physician	1 (10%)	5 (33%)
Surgeon	6 (60%)	10 (67%)
**Department**		
Accident and Emergency	1 (10%)	2 (13%)
Anaesthesia	1 (10%)	2 (13%)
Burns and Plastic Surgery	0 (0%)	1 (6.7%)
Critical care	1 (10%)	0 (0%)
Hospital administration	1 (10%)	0 (0%)
Orthopedic surgery	1 (10%)	2 (13%)
Radiology	0 (0%)	1 (6.7%)
Surgery	5 (50%)	7 (47%)
**Female/Male ratio**		
Female	4 (40%)	3 (20%)
Male	6 (60%)	12 (80%)

[1]n (%)

The online surveys, consensus and stability criteria

We used the online survey software, LimeSurvey, to create an anonymous survey for each site.[14] Potential participants were invited to a website with information about the project, where they could register and then take part in the survey.

The audit filters were presented to the participants without revealing their sources. Rating of the perceived usefulness, defined as usefulness from the perspective of experts, was

done on an ordinal Likert scale with grades from 1-10. A score of 1 represented not useful and 10 very useful. Open comments could be entered for each filter, and new filters could be proposed. Modifications to the filters could also be suggested. Comments were reviewed and summarized by members of the core research team after completion of each round; these were presented along with the median score of the filter in subsequent rounds.

Filters that received a median score of 7 or higher and newly added filters were included in subsequent rounds. Filters with a score of <7 were immediately rejected. A filter was deemed complete when it was either rejected or reached consensus for the selection, defined as having a median score of 9 or higher over two rounds, or when stable, defined as no significant changes in responses between rounds. A stable filter with a score of <9 was rejected. Stability was determined using the Wilcoxon-rank-sum test, comparing scores from the previous and current rounds for each filter. A significance level of 0.05 was used. Filters modified based on comments from the panellists were included in subsequent rounds even if they had previously reached a consensus. The iterations continued until all remaining filters reached either consensus or stability. Filters that had a median score of 9 and above were selected for implementation at each site. Reminders were sent via email up to three times if no reply had been recorded.

Statistical analysis

To compare the perceived usefulness between different sources of audit filters, the filters were categorized into three groups based on their origin (LMIC, WHO, and New). The filters defined in Cameroon and Ghana were combined as the group LMIC, the WHO guidelines as WHO, and new filters proposed by the panellists at the sites as New. Filters that were modified were still included in the group of their origin.

The score from all participants in the final round for each filter was used for analysis. For example, if a filter was rejected in round 2, the scores from all participants in that round were used for analysis. If a filter was selected in round 3, scores from that round and filter were used. We used the Kruskal-Wallis test to compare usefulness scores across the three groups. If this resulted in a significant difference ($p<0.05$), further analysis was conducted using the Wilcox-rank-sum test to find differences between the individual groups using a significance level of $p<0.05$. We used the Bonferroni method to adjust p-values for multiple tests. We used R for all analyses.[15]

Content analysis

We explored the characteristics of the audit filters that were discussed using inductive content analysis.[16] We collated the comments from both sites. For the initial analysis, the comments were organized so that only the comments and not the associated filters were visible. We did this to detach the comments from the filters and focus on the general problems or characteristics discussed. First, the entire collection of comments was read several times. Then meaning units were identified and abstracted to codes. All codes were sorted into categories that were abstracted into themes. After this, we assessed how the themes and categories compared to usefulness scores and the defined groups. We did this by selecting a theme or a category, looking at which group of filters these were derived

For peer review only

from to see if one group was more frequently represented than another. If this was the case, we went back to the comments associated with this theme and group to understand the specific discussion better. We used this frequency analysis to help find patterns in the comments that would have been difficult to detect. We used the NVivo software for analysis.[17]

Results

Surveys

The online surveys were performed from December 2018-March 2019. A total of four rounds were done at each site. The response rates remained stable with high participation rates for each round (Site one: 80-100%, Site two: 93%). In total, 30 filters for site one and 37 filters for site two were processed, resulting in a selection of 24 and 32 filters, respectively. (Fig: 1)

For peer review only

Perceived usefulness

The LMIC filters had the highest mean usefulness score at both sites *(Site one: 9.132, site two; 9.568 of 10)*. Comparing all three groups, we found significant differences in usefulness scores at both sites *(Kruskal-Wallis test: Site one p-value<0.001, site two p-value<0.001).* Comparing pairwise between groups at each site, we found significant differences in usefulness scores between all groups, except between New and WHO filters at site one. *(p-value=1)*. (Fig: 2) Filters that were modified during rounds had no significant difference in scores compared to those that were not modified. *(Site one: p=0.322. Site two: p=0.067.)* (Table 2)

For peer review only

Table 2: Processed audit filters, rejected filters highlighted in red

Group	ID	Audit filter	Score	Site	Modified
WHO	3.1	Hourly GCS in the emergency department of trauma patients with a diagnosis of skull fracture, intracranial injury or spinal cord injury	10.0	1	Yes
	3.2	Sequential (every 30 minutes) GCS of trauma patients with a diagnosis of skull fracture, intracranial injury or spinal cord injury	10.0	2	Yes
	5	Documentation of history and physical examination by a doctor	10.0	1	No
			10.0	2	No
	6.1	Head computerized tomography (CT) scan done within 2 hours of arrival at hospital for a non-transferred patient with Glasgow Coma Scale score <13 and systolic blood pressure > 90	6.5	1	No
	6.2	Head computerized tomography (CT) scan done within 2 hours of arrival at hospital for a non-transferred patient with Glasgow Coma Scale score < 8 and systolic blood pressure > 90	10.0	2	Yes
	14	Operative treatment of gunshot wound to the abdomen	9.0	1	No
			10.0	2	No
	15.1	Fixation of femoral fracture in adult patient within 24 hours of arrival to emergency department	6.5	1	No
	15.2	Fixation of isolated closed femoral shaft fracture in adult patient within 24 hours of arrival to emergency department	7.0	2	Yes
LMIC	21.1	Vital signs recorded within 5 minutes of arrival to emergency department (must include breathing assessment, heart rate, blood pressure, oxygen saturation if available)	10.0	1	Yes
	21.2	Vital signs recorded within 5 minutes of arrival to emergency department (must include breathing assessment, heart rate, blood pressure, oxygen saturation).	10.0	2	Yes
	22	Senior medical officer made aware of patient with difficulty breathing, or shock present at triage (HR >100, OR SBP <110)* or oxygen saturation <95% within 5 minutes of initial assessment	10.0	1	No
			10.0	2	No
	23	The clinician did assess airway patency by asking the patient a question and listening for a response	10.0	1	No
			10.0	2	No
	24.1	Basic airway manoeuvre assistance (i.e. sweep, chin-lift-jaw-thrust, oral or nasal airway, suction) performed for patient with difficulty or obstructed breathing.	10.0	1	No
	24.2	Basic airway manoeuvre assistance (i.e. jaw-thrust, oral or nasal airway, suction, removal of foreign object) performed for patient with difficulty or obstructed breathing.	10.0	2	Yes
	25	Examination for pneumo- hemothorax done by listening to both sides of the chest with a stethoscope within 5 minutes of patient arrival to emergency department	10.0	1	Yes
			10.0	2	Yes
	26	Chest tube placed within 30 min of patient arrival in patient with suspected or confirmed pneumo- or hemothorax and oxygen saturation less than 98%	9.0	1	No
			10.0	2	No
	27.1	Large bore IV was placed within 5 minutes of patient arrival to the emergency department	10.0	1	Yes
	27.2	Large bore IV was placed within 5 min of patient arrival to the emergency department in patients with tachycardia (heart rate > 110) or hypotension (systolic blood pressure < 90)	10.0	2	Yes
	28	Pressure applied to external bleeding at patient arrival to the emergency department, and maintained until definitive control is performed	10.0	1	No
			10.0	2	No
	30.1	Reduction and/or splinting with analgesia made for a long bone fracture within 2 hours of admission or prior to transfer	10.0	1	No

Group	ID	Audit filter	Score	Site	Modified
	30.2	Splinting with analgesia made for a long bone fracture within 30 minutes of admission or prior to transfer	10.0	2	Yes
	35.1	Burn patient did receive 2-4 mL of crystalloid solution per kilogram body weight per percent body surface burn within 24 hours of injury	10.0	1	No
	35.2	Burn patient did receive 4 mL of Ringer's lactate per kilogram body weight per percent body surface burn within 24 hours of injury	10.0	2	Yes
	36.1	Senior attending physician alerted when airway is compromised, usage jaw thrust, chin lift, ORA/NPA, or suction to open airway	10.0	1	No
	36.2	Senior attending physician alerted within 5 minutes of patient arrival to the emergency department when airway is compromised, usage jaw thrust, chin lift, ORA/NPA, or suction to open airway	10.0	2	Yes
	37	Assessment of mouth/throat for foreign bodies and debris made in patient that has difficulty breathing, within 10 minutes of arrival to emergency department	10.0	1	No
			10.0	2	No
	38.1	Breathing assessment made within 15 min of arrival to emergency department	10.0	1	No
	38.2	Breathing assessment made within 5 min of arrival to emergency department	10.0	2	Yes
	40.1	Patient assessed for hypovolemia when presenting with hypotension and tachycardia or suspected intra-abdominal bleeding, femoral shaft fracture, or pelvic fracture.	10.0	1	No
	40.2	Patient assessed for hypovolemia using clinical examination, USG, FAST, or DPL within 15 minutes of arrival to the emergency department when presenting with hypotension and tachycardia or suspected intra-abdominal bleeding, femoral shaft fracture, or pelvic fracture.	10.0	2	Yes
	45.1	Laparotomy done within 1 hour of arrival to the emergency department in a patient with abdominal injuries and systolic blood pressure <90	5.5	1	No
	45.2	Laparotomy done within 1 hour of arrival to the emergency department in a patient with abdominal injuries and systolic blood pressure <90 after fluid resuscitation	10.0	2	Yes
	46.1	Immobilization and imaging performed in a patient with suspected spine injury, within 4 hours of arrival to the emergency department	10.0	1	No
	46.2	Immobilization within 10 minutes and imaging performed within 4 hours of arrival to the emergency department in a patient with suspected spine injury	10.0	2	Yes
	47	I.v antibiotics given within 1 hour of arrival to the emergency department in a patient with an open fracture	10.0	1	No
			10.0	2	No
	49.1	Operation for irrigation and debridement within 12 hours from arrival to emergency department for an open fracture	10.0	1	No
	49.2	Operation for irrigation and debridement within 6 hours from arrival to emergency department in a hemodynamically stable patient with an open fracture	10.0	2	Yes
	58.1	Intubation performed in patient with a GCS score of 8 or less within 30 minutes of arrival to emergency department.	10.0	1	No
	58.2	Intubation performed in patient with a GCS score of 8 or less within 10 minutes of arrival to emergency department.	10.0	2	Yes
	62	Operation for sub or epidural hematoma within 3 hours of arrival to emergency department	8.0	1	No
			9.0	2	No
	65	FAST exam performed within 30 minutes from arrival to the emegency department to exclude hemoperitoneum.	10.0	1	No
			9.0	2	No

Group	ID	Audit filter	Score	Site	Modified
New	66.1	Antibiotics used in acute major (50% or more) burns within 24 hrs	6.0	1	NA
	66.2	Response time of respective department in attending the call	9.0	2	NA
	67.1	MESS or WHO Trauma scale used in prognosis mangled upper extremity	9.0	1	NA
	67.2	Sample sent for investigations	8.0	2	NA
	68.1	Facial 3 D scan done to rule out facial fractures within 24 hours of arrival to the emergency department	5.0	1	NA
	68.2	Airway breathing and circulation assessed immediately on arrival of patient to the emergency department	10.0	2	NA
	69.1	Serial assessment of vitals and GCS after admission	10.0	1	NA
	69.2	Oxygen therapy with simple face mask initiated in a patient whose SpO2 is less than 92% within 5 minutes of initial assessment of the patient	10.0	2	NA
	70	Response time in initiating definite treatment from arrival to the emergency department, by specialist department, within 1 hour from arrival to the emergency department	9.0	2	NA
	71	Blood components started within 4 hours of arrival to the emergency department if the patient has a Hb < 7g/dl.	9.0	2	NA
	72	AVPU for initial assessment and followed by sequential GCS	9.0	2	NA
	73	Response time of surgeons	8.0	2	NA
	74	Ionotropes in patient of shock	6.0	2	NA
	75	Drug assisted intubation	6.5	2	NA
	76	Sample sent for blood group and cross match in patients with significant bleeding (heart rate > 110 or systolic blood pressure < 90) within 15 minutes of arrival to the emergency department	10.0	2	NA

Content analysis

Three major themes relating to an audit filter's feasibility, medical relevance and specificity evolved. Creating audit filters appears to be a balance between medical relevance and feasibility as well as generality and specificity. Logistic limitations may force providers to modify standards of care to make something feasible. For example, if there is limited availability to an operation theatre (OT), someone needs to prioritize which patient should get access first. Defining this in audit filters is a balance between medical relevance (the most injured patient goes to OT first) and feasibility (Limited to one OT, it might not be feasible to state that non-life-threatening injuries should have surgery within 6 hours). The filters need to be general enough to be valid for a substantial part of the patient population but specific enough to state what interventions need to be done, when they are to be done, and which patients. All themes and high-level subcategories are in table 3.

Table 3: Identified themes and subcategories.

Themes
**Theme 1 Assessing medical relevance**
Filter deemed medically important
Modifying filters based on a medical rationale
Questions medical relevance or correctness

Themes
**Theme 2 Questioning feasibility**
Concern that blame will be on the provider
Filter describes something ideal - but not achievable
Logistic limitations
Lacking equipment
Lacking ICU beds
Lacking operation theatre
Lacking staff
Timeframe not feasible
Modifying filters to make them more achievable
Make the filter more conservative
Limiting interventions only for patients that need it
Task shifting
Everyone on the trauma team can do simple interventions
**Theme 3 Increasing filter specificity**
Argues for higher specificity of the filter
Defining cutoffs
Defining target population
Defining time to intervention
Defining wherein the process filter should apply
Defining who should do what

Assessing medical relevance

Filters perceived as medically relevant received high usefulness scores, and filters not perceived as medically relevant received lower scores. This may indicate that one of the main characteristics of an audit filter, for it to be perceived as useful, is that it is perceived as medically relevant. If considered relevant the filters could score highly, even if they had difficult feasibility problems. Filters that originated from WHO was to a higher extent discussed as being less medically relevant than filters from LMIC sources.

For example, discussions on filter 65 **"FAST exam performed within 30 minutes from arrival to the emergency department to exclude hemoperitoneum."**

"Subject to availability (remark: of the ultrasound machine), may be dropped at the moment…"

"FAST should stay as an audit filter as it's integral."

"Don't attempt to reframe this extremely useful filter. Instead channel energies to get FAST for emergency."

In the above example, there is a difficult feasibility problem, the lack of ultrasound machines. However, there was agreement that this intervention is so medically relevant that the filter should be implemented anyway and be used to advocate for ultrasound resources in the emergency department.

Feasibility

In some instances, participants agreed that filters described something medically ideal but not deemed feasible. Reasons for this were lack of resources such as equipment or staff or feasibility in data collection.

For example, while discussing filter 62, **"Operation for sub or epidural hematoma within 3 hours of arrival to emergency department",** panellists commented:

"Feasibility of performing emergency operation within the desired time frame of 3 hours of arrival for this condition remains an issue."

On occasion, if the filter was deemed medically important, it was modified to make it more achievable. This could be done by restricting the patient population that should receive the intervention or prolonging the time to intervention while balancing this towards the medical relevance of the intervention. For example, filters that describe simple airway manoeuvres should be done immediately, whereas a filter stating time to CT may be prolonged.

For example, filter 6 was originally **"Head computerized tomography (CT) scan done within 2 hours of arrival at hospital for a non-transferred patient with Glasgow Coma Scale score <13 and systolic blood pressure > 90".** At one site this was modified from a cutoff of GCS < 13 to GCS < 8 causing a significant change in the target population, increasing the likelihood of significant pathology and reducing the intended use of CT. After this, the filter received high scores and reached a consensus.

Filter specificity

The discussions within this theme reflected that filters could have a varying degree of specificity, that is, how precise they were in defining a target population, an intervention and often a time to that intervention. These cutoffs, to define the target population and time to intervention, were heavily discussed. The cutoffs may not be backed by a high degree of medical evidence and hence become based on consensus.

Example, filter 27: **"Large bore IV was placed within 5 min of patient arrival to the emergency department in patients with tachycardia (heart rate > 110) or hypotension (systolic blood pressure < 90)"**
This filter was agreed upon after four iterations. It contains several cutoffs, one that defines a time to intervention (within 5 minutes), and two thresholds defining the target population should receive the intervention (Patients with HR > 110 or SBP < 90).

This theme intersects with the theme of making filters feasible for implementation because making a filter more conservative may limit the number of patients exposed to an intervention or allow for more time to intervention, making the filter feasible in this

For peer review only

context. However, agreeing on specific limits and framing to increase the specificity of the filter was not always done to make it more feasible.

Filter specificity was heavily discussed for filters with high and low usefulness scores within all groups, indicating that specificity is a general characteristic of an audit filter and may not be what deems it as being useful.

Discussion

We found that the filters defined in LMIC had significantly higher usefulness scores and were deemed highly medically relevant compared with filters from the WHO. One reason for this may be that the filters suggested by WHO are derived mainly from filters in mature trauma systems in HIC.[7][8] Filters from LMIC may be reflective of trauma systems more similar to the urban India context.

The American College of Surgeons Committee on Trauma (ACSCOT) published the first audit filters for trauma care improvement in 1987. These 12 filters were derived by expert opinion, and since then, they have evolved and been applied in several trauma systems. [8] Over time it has become clear that filters need to be adapted to local environments, and since 2006, ACSCOT does not list specific filters but recommends filter tracking depending on local priorities. The WHO guidelines also emphasize that the filters listed are potential and that their usefulness may depend on local circumstances.[7]

The audit filters defined in Cameroon [9] and Ghana [10] were deemed highly useful in the urban Indian setting. The filters from Cameroon were focused on regional referral hospitals, while those defined in Ghana were intended for use in district-level hospitals. Many of these filters represent the initial management, resuscitation and simple, potentially life-saving interventions. These filters may not be perceived as useful in a mature trauma system where these interventions are assumed.

Several studies from HIC have struggled to determine the effect on outcomes of specific audit filters. The review process itself, being costly and labour-intensive, has raised questions about the usefulness of audit filters in well-developed trauma systems.[8,18] Their potential use in less developed trauma systems is less studied but seems promising. One study by Chadbunchachai et al. in Thailand showed a significant reduction of preventable trauma deaths between 1994 and 1997 after implementing trauma audit filters.[5,19] The discrepancy that the same filter may be useful and potentially influence outcomes in one context and not the other highlights the importance of audit filters being selected and evolved locally to suit the context of their use.

Hypothetically, applying filters developed in HIC and mature trauma systems to a low-resource setting may be disruptive since they may divert focus from the more pressing concerns and hence not improve the processes that could affect outcomes in that context.

The LMIC definition is broad; we have chosen to use it here as a distinction against HIC with mature trauma systems. Cameroon, Ghana and India would be classified more specifically as lower-middle-income countries. For low-income countries, there is very

For peer review only - http://bmjopen.bmj.com/site/about/guidelines.xhtml

limited research on trauma systems.[4] Whether the filters evaluated in this study would be transferable to low-income countries is unclear. Treatment of a majority of trauma patients requires a health care infrastructure that can supply surgical services. This is lacking in several contexts, especially in low-income, lower-middle-income countries and in rural areas.[20]

We found that the new suggested filters at both sites had the lowest median scores. This may reflect the difficulty of defining audit filters based on consensus with online survey interaction only. Developing new audit filters that are clear, feasible and specific is a complex task. If this is attempted via an online survey, it may be beneficial to supply the participants with a short suggestion on how an audit filter may be formulated to make sure it is specific, highlights the medical relevance and is feasible in the context.

One of this study's main strengths is the nesting within the TAFT project since both sites performed the surveys to select audit filters for implementation in the hospital. The use of two separate sites adds strength to our results. The online Delphi approach enabled us to execute a study at two sites in different cities, with a wide range of participants at a very low cost. Similar studies could be replicated in other low-resource settings with standard technical devices.

There are several limitations to our study. Firstly, the pre-selection of audit filters made by the core team may induce a bias and may have contributed to the overall high usefulness scores of the filters. However, analysis of the results of this pre-selection Delphi did not reveal any significant difference in usefulness scores between filters from WHO and LMIC during the pre-selection. (Supplemental material: Figure 1) The actual number of filters from the WHO was also lower, and one filter receiving low scores had a large impact overall. The statistical analysis is based on a convenience sample, not a truly randomized sample, but we believe that the significance testing is still of value. The overall high scores contribute to a ceiling effect which may affect our results.

Our expert panels mainly consisted of surgeons and physicians, and perspectives from other health care personnel were limited. Management of the trauma patient involves a broad team of personnel and including panellists who reflect this is important for future studies in this area.

The content analysis was done based on comments, not by directed questions. It was not mandatory to leave a comment, and hence this discussion primarily focuses on problems or features to improve. Since the main focus of the discussion is to agree to something standardized and measurable, very little about experiences or deeper mechanisms can be extracted from this material. We believe, however, that the content analysis gives another level of understanding as to why filters received the scores that they did, which may help guide efforts when adapting audit filters in other contexts.

Conclusion

The trauma audit filters defined in Ghana and Cameroon were deemed highly useful in the urban India context, indicating that the potential for transferability of trauma audit filters

For peer review only

between similar contexts is high. These filters may be used as a starting ground when implementing trauma improvement programs including audit filters in similar settings. Compared to filters derived from high-resource programs, many of these filters focus on initial interventions and resuscitation and were deemed to have high medical relevance in this context.

For peer review only

Acknowledgements

We would like to thank all the panellists in the Delphi survey for their time and dedication to this project. Their constructive work in refining the audit filters is what made this study possible. A warm thank you to the participating centres, where the efforts by a great many continue as these filters are implemented and continuously used for the review. Finally, thank you to all the participants in the ongoing Trauma Audit Filter Trial: clinicians, health care staff, our project officers and mostly all patients and relatives that generously allow us to include them.

Contributorship statement

MGW, NR, KDS, MK conceived the study as part of the Trauma Audit Filter Trial. JB, MGW, HMA, NR, KDS, MK contributed to the design of the study. MGW, NR, KDS, MK and SD conducted the on-site training before the online Delphi. JB, MGW, NR, LB, PKB, SC, SD, SG, MK, PL, VM, RM, AM, LNM, RS, PS, RS, KDS and SS were a part of the execution of the online Delphi. JB prepared the data. JB and MGW conducted the statistical analysis. MP reviewed the statistical analysis. JB performed the content analysis. JB and MGW drafted the first version of the article. All authors contributed to the refinement of the article.

Competing interests

None declared.

Funding

This work was supported by the Swedish Research Council (2016-02041).

Data sharing statement

Data and R-code for the quantitative analysis are publicly available on GitHub at https://github.com/titco/Usefulness-of-trauma-audit-filters. To protect the privacy of the participants the qualitative data is not available.

Figure 1: Delphi iterations and filter selection

Figure 2: Usefulness scores and level of significance for trauma audit filters by source group

For peer review only

References

1 GBD 2017 Causes of Death Collaborators. Global, regional, and national age-sex-specific mortality for 282 causes of death in 195 countries and territories, 1980-2017: a systematic analysis for the Global Burden of Disease Study 2017. *Lancet (London, England)* 2018;**392**:1736–88. doi:10.1016/s0140-6736(18)32203-7

2 Kotagal M, Agarwal-Harding KJ, Mock C, *et al.* Health and Economic Benefits of Improved Injury Prevention and Trauma Care Worldwide. *PLoS ONE* 2014;**9**:e91862. doi:10.1371/journal.pone.0091862

3 Juillard CJ, Mock C, Goosen J, *et al.* Establishing the Evidence Base for Trauma Quality Improvement: A Collaborative WHO-IATSIC Review. *World journal of surgery* 2009;**33**:1075–86. doi:10.1007/s00268-009-9959-8

4 Reynolds TA, Stewart B, Drewett I, *et al.* The Impact of Trauma Care Systems in Low- and Middle-Income Countries. *Annual review of public health* 2017;**38**:507–32. doi:10.1146/annurev-publhealth-032315-021412

5 The International Society of Surgery (ISS) and the G4 Alliance International Standards and Guidelines for Quality Safe Surgery and Anesthesia (ISG-QSSA), Jin J, Akau'ola S, *et al.* Effectiveness of Quality Improvement Processes, Interventions, and Structure in Trauma Systems in Low- and Middle-Income Countries: A Systematic Review and Meta-analysis. *World Journal of Surgery* 2021;1–7. doi:10.1007/s00268-021-06065-9

6 Evans C, Howes D, Pickett W, *et al.* Audit filters for improving processes of care and clinical outcomes in trauma systems. *Cochrane Database of Systematic Reviews* 2009;**84**:782. doi:10.1002/14651858.cd007590.pub2

7 World Health Organization. *Guidelines for trauma quality improvement programmes*. 2009. https://apps.who.int/iris/handle/10665/44061

8 Ronald Maier, Michael Rhodes. Trauma Performance Improvement. In: *Injury control: A guide to research and program evaluation*. Cambridge: : Cambridge University Press 236–49. doi:DOI: 10.1017/CBO9780511530234.017

9 Wu N, Carvalho M, Nwanna-Nzewunwa O, *et al.* Developing Trauma Audit Filters for Regional Referral Hospitals in Cameroon: Mixed-Methods Approach. *The Journal of surgical research* 2018;**232**:578–86. doi:10.1016/j.jss.2018.07.048

10 Stewart BT, Gyedu A, Quansah R, *et al.* District-level hospital trauma care audit filters: Delphi technique for defining context-appropriate indicators for quality improvement initiative evaluation in developing countries. *Injury* 2016;**47**:211–9. doi:10.1016/j.injury.2015.09.007

11 Roy N, Gerdin M, Ghosh S, *et al.* 30-Day In-hospital Trauma Mortality in Four Urban University Hospitals Using an Indian Trauma Registry. *World Journal of Surgery* 2016;**40**:1299–307. doi:10.1007/s00268-016-3452-y

For peer review only

12 Roy N, Veetil DK, Khajanchi MU, *et al.* Learning from 2523 trauma deaths in India-opportunities to prevent in-hospital deaths. *BMC Health Services Research* 2017;**17**:142. doi:10.1186/s12913-017-2085-7

13 Hsu C-C, Sandford BA. The Delphi Technique: Making Sense Of Consensus. *Practical Assessment, Research & Evaluation* 2007;**12**. doi:doi.org/10.7275/pdz9-th90

14 GmbH. LimeSurvey: An Open Source survey tool. 2006.https://www.limesurvey.org/

15 R Foundation for Statistical Computing. R: A language and environment for statistical computing. https://www.r-project.org/

16 Krippendorff K. *Content analysis : an introduction to its methodology*. Fourth edition. SAGE 2018.

17 QSR International. NVivo Qualitative Data Analysis Software. 2020.https://www.qsrinternational.com/nvivo-qualitative-data-analysis-software/home

18 Willis CD, Gabbe BJ, Cameron PA. Measuring quality in trauma care. *Injury* 2007;**38**:527–37. doi:10.1016/j.injury.2006.06.018

19 Chadbunchachai W, Sriwiwat S, Kulleab S, *et al.* The comparative study for quality of trauma treatment before and after the revision of trauma audit filter, Khon Kaen hospital 1998. *Journal of the Medical Association of Thailand = Chotmaihet thangphaet* 2001;**84**:782–90.

20 Meara JG, Leather AJM, Hagander L, *et al.* Global Surgery 2030: evidence and solutions for achieving health, welfare, and economic development. *The Lancet* 2015;**386**:569–624. doi:10.1016/s0140-6736(15)60160-x

For peer review only

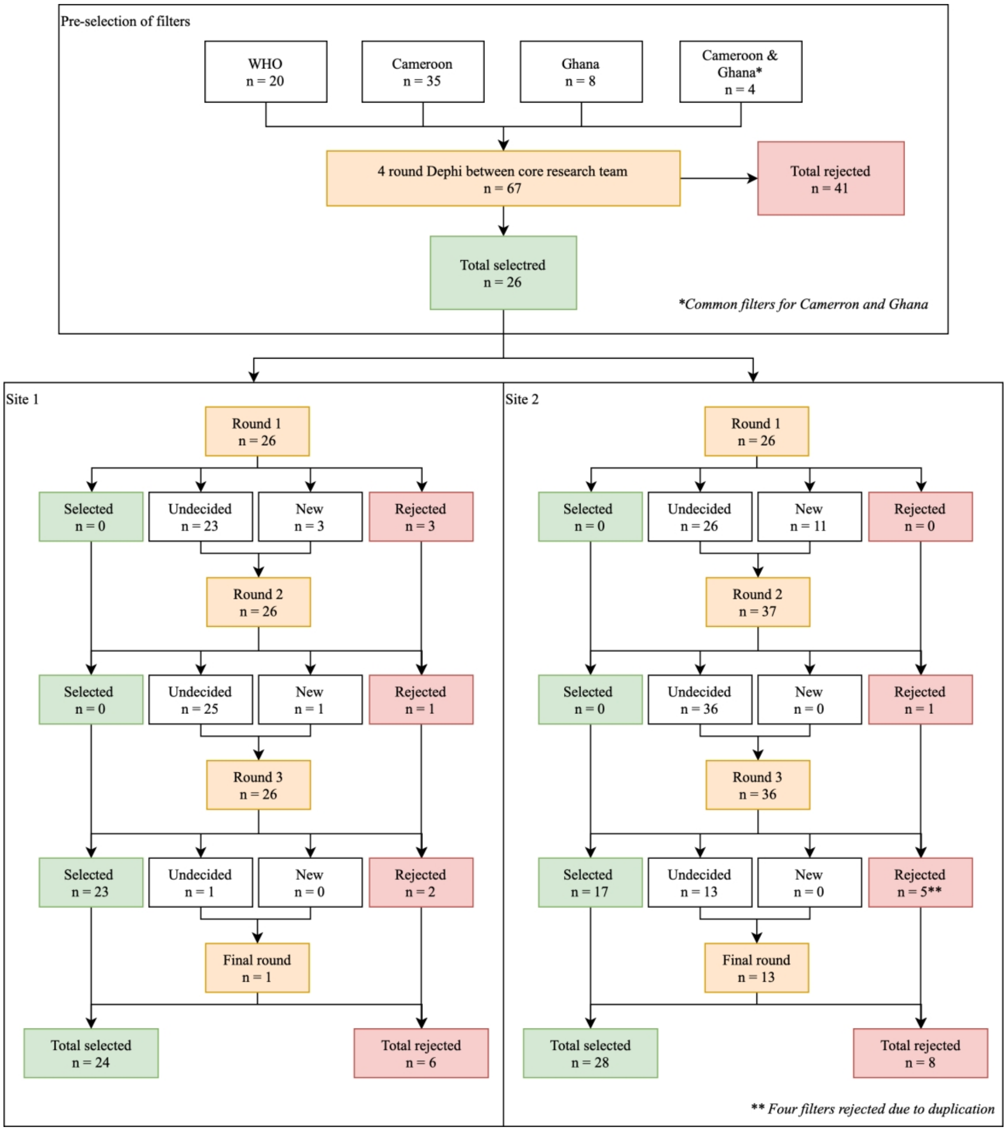

Figure 1: Delphi iterations and filter selection

530x594mm (118 x 118 DPI)

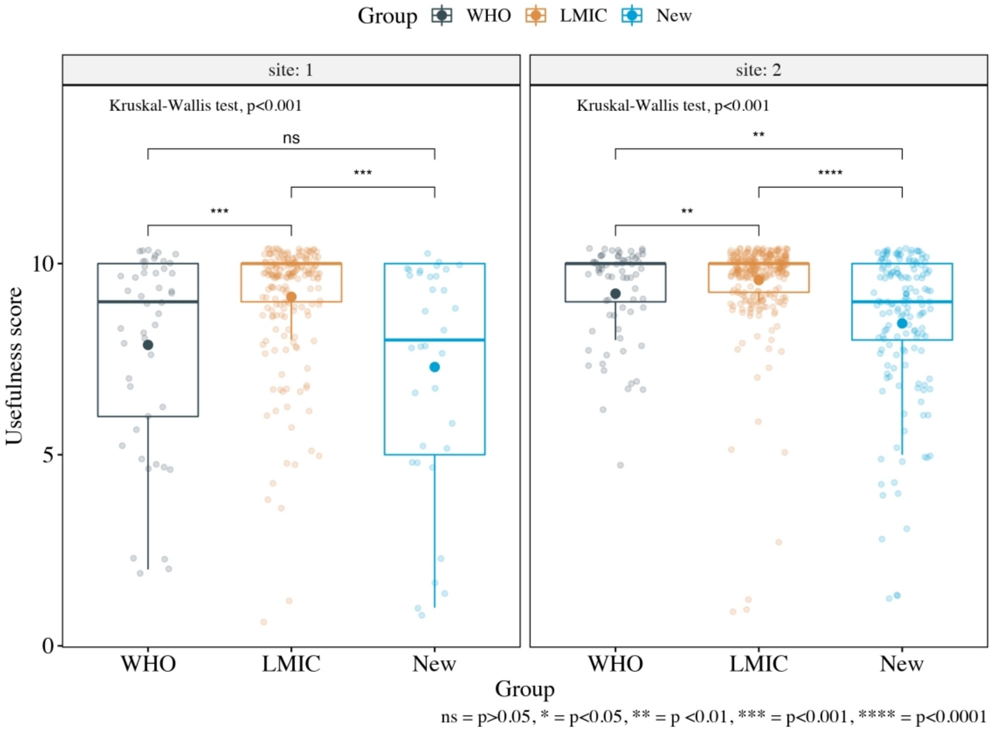

Figure 2: Usefulness scores and level of significance for trauma audit filters by source group

610x457mm (118 x 118 DPI)

Supplemental material

11/27/2021

Supplemental material

Analysis of the pre-Delphi trial

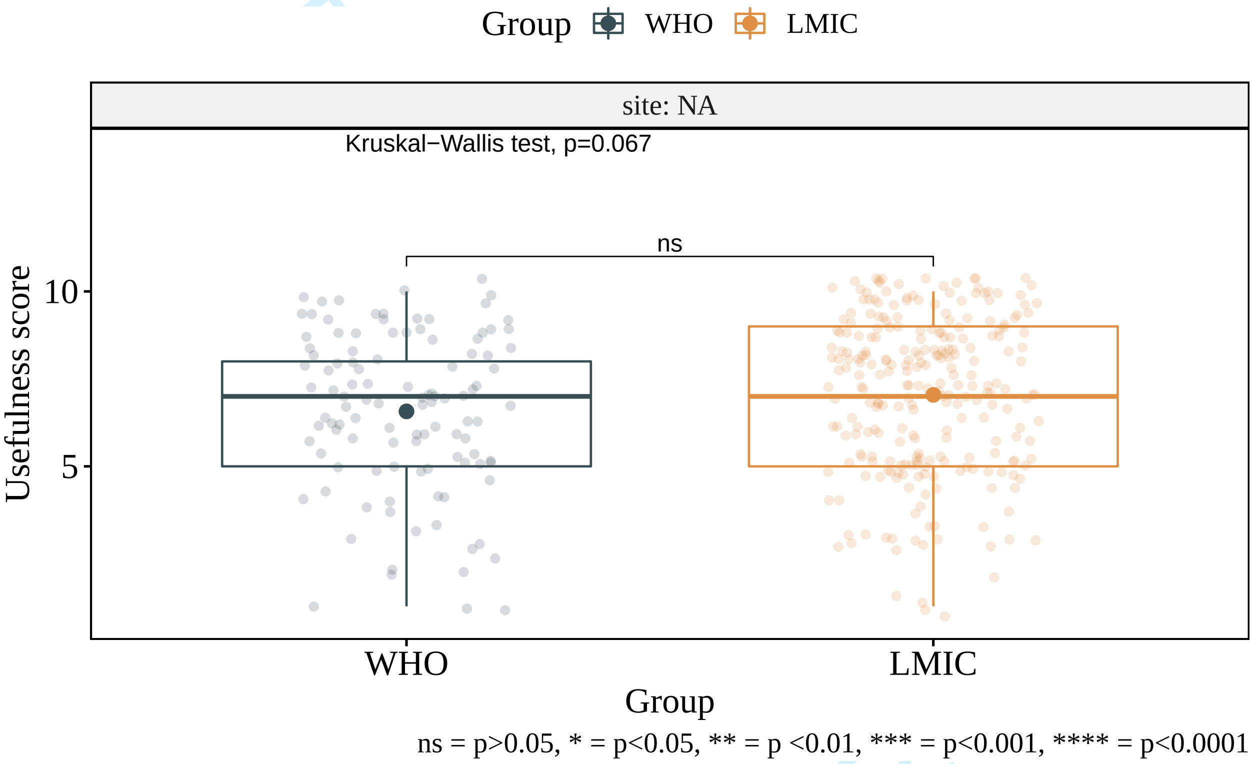

Figure 1: Pre-Delphi: Usefulness scores and level of significance for trauma audit filters by source group

For peer review only - http://bmjopen.bmj.com/site/about/guidelines.xhtml

All initial audit filters

Table 1: All initial audit filters. n = 67

Group	ID	Audit filter
WHO	1	Response time of surgeons
WHO	2	Availability of operating room
WHO	3	Sequential GCS in the emergency department of trauma patients with a diagnosis of skull fracture, intracranial injury or spinal cord injury
WHO	4	At least hourly, determination and recordings of blood pressure, pulse, respirations, temperature, Glasgow Coma Scale (GCS) score and intake and output (I&O) measurements for a major or severe trauma patient done. Beginning with arrival in the resuscitation area and including time spent in radiology up to admission to the operating room or ICU, death, or transfer to another hospital
WHO	5	Documentation of history and physical examination by a doctor
WHO	6	Head computerized tomography (CT) scan done within 2 hours of arrival at hospital for a non-transferred patient with Glasgow Coma Scale score <13
WHO	7	Endotracheal tube or surgical airway performed before leaving resuscitation area in patient with Glasgow Coma Scale score <8
WHO	8	No laparotomy within 1 hour of arrival at the hospital for patient with abdominal injuries and hypotension (systolic BP <90)
WHO	9	Laparotomy not performed (within 4 hours to or 24 hours after admission depending on hospital practice)
WHO	10	Craniotomy performed within 4 hours of arrival to the emergency department, for drainage of epidural or subdural haematoma
WHO	11	Abdominal, thoracic, vascular or cranial surgery after 24 hours
WHO	12	Unplanned return to operating theatre within 48 hours of initial procedure
WHO	13	Patient requiring re-intubation of the airway within 48 hours of extubation
WHO	14	Operative treatment of gunshot wound to the abdomen
WHO	15	Fixation of femoral fracture in adult patient within 24 hours of arrival to emergency department
WHO	16	All delays in identification of injuries
WHO	17	All trauma deaths
WHO	18	Required equipment, shared with other departments (e.g. fluid warmer, ventilator), not immediately available when requested
WHO	19	Non-compliance with institutional protocols
WHO	20	All major complications (e.g. deep venous thrombosis, pulmonary embolus, decubitus ulcers
Ghana and Cameroon	21	Vital signs recorded within 15 minutes of arrival (must include breathing assessment, heart rate, blood pressure, oxygen saturation if available)
Ghana	22	Senior medical officer made aware of patient with difficulty breathing, OR shock present at triage (HR >100, OR SBP <110)* OR oxygen saturation <95% within 5 minutes of initial assessment
Ghana	23	The clinician did assess airway patency by asking the patient a question and listening for a response
Ghana	24	Basic airway manoeuvre assistance (i.e. sweep, chin-lift-jaw-thrust, oral or nasal airway, suction) performed for patient with difficulty or obstructed breathing.
Ghana	25	Examination for pneumo- hemothorax done by listening to both sides of the chest with a stethoscope and bilateral percussion, within 60 minutes of patient arrival to emergency department

For peer review only - http://bmjopen.bmj.com/site/about/guidelines.xhtml

Group	ID	Audit filter
**Ghana**	26	Chest tube placed within 30 min of patient arrival in patient with suspected or confirmed pneumo- or hemothorax and oxygen saturation less than 98%
**Ghana**	27	Large bore IV was placed within 15 min of patient arrival to the emergency department
**Ghana and Cameroon**	28	Pressure applied to external bleeding at patient arrival, and maintained until definitive control is performed
**Ghana and Cameroon**	29	If AVPU is not 'A' AND the patient is not in shock, the head of the bed is elevated to 45 degrees.
**Ghana**	30	Reduction and/or splinting with analgesia made for a long bone fracture within 2 h of admission or prior to transfer
**Ghana and Cameroon**	31	Patient is completely undressed, fully examined and covered for privacy within 30 min of arrival
**Ghana**	32	Fluid order for a burn patient not done using the Parkland formula is recorded within 1 h for burns over 15% total body surface area that occurred less than 24 h from patient arrival
**Cameroon**	33	No sufficient history from patient or next best historian obtained.
**Cameroon**	34	No emergency department team prepared and ready with sufficient staff before patient arrival.
**Cameroon**	35	Burn patient did not receive 2-4 mL of crystalloid solution per kilogram body weight per percent body surface burn within 24 h of injury
**Cameroon**	36	Senior attending physician alerted when airway is compromised, usage jaw thrust, chin lift, ORA/NPA, or suction to open airway
**Cameroon**	37	Assessment of mouth/throat for foreign bodies and debris made in patient that has difficulty breathing, within 10 minutes of arrival to emergency department
**Cameroon**	38	Breathing assessment made within 15 min of arrival to emergency department
**Cameroon**	39	Establish two large bore IVs within 15 min of arrival.
**Cameroon**	40	Patient assessed for hypovolemia when presenting with hypotension and tachycardia or suspected intra-abdominal bleeding, femoral shaft fracture, or pelvic fracture.
**Cameroon**	41	Neurological assessment made with AVPU.
**Cameroon**	42	If patient arrived on spinal board, when appropriate patient is subsequently safely logrolled off board.
**Cameroon**	43	Open wounds are treated and covered
**Cameroon**	44	Chest X-ray performed for pneumothorax, hemothorax, and flail chest diagnosis.
**Cameroon**	45	Laparotomy not done within 1 h of arrival to hospital in a patient with abdominal injuries and systolic blood pressure <90
**Cameroon**	46	Immobilization and imaging performed in a patient with suspected spine injury, within 4 hours of arrival to the emergency department
**Cameroon**	47	I.v antibiotics given within 1 hour of arrival to the emergency department in a patient with an open fracture
**Cameroon**	48	Long bone fracture is reduced with analgesia and/or splinted within 4 h of admission or prior to transfer.
**Cameroon**	49	Operation for irrigation and debridement within 12 h from arrival to emergency department for an open fracture
**Cameroon**	50	Before patient arrival, emergency department is adequately stocked with essential supplies.
**Cameroon**	51	Referral made within 15 min of determination if treatment of injury is beyond site's capabilities (i.e. not able to operate).
**Cameroon**	52	Second surgery within 48 h of previous operation

For peer review only

Group	ID	Audit filter
**Cameroon**	53	Unplanned return to operating theater.
**Cameroon**	54	Unplanned intubation.
**Cameroon**	55	Unplanned return to intensive care unit.
**Cameroon**	56	Unplanned readmission.
**Cameroon**	57	No ventilation done with bag-valve mask in patient who can not self ventilate.
**Cameroon**	58	Intubation performed in patient with a GCS score of 8 or less.
**Cameroon**	59	Thoracostomy within 30 min of arrival to emergency department in patient with pneumothorax or haemothorax
**Cameroon**	60	Patient body temperature is maintained by giving warmed IV fluids and blood to prevent hypothermia
**Cameroon**	61	If intracranial pressure <20 mm Hg, patient treated with mannitol or hypertonic saline
**Cameroon**	62	Operation for sub or epidural hematoma within 3 hours of arrival to emergency department
**Cameroon**	63	No FAST exam performed to evaluate for pericardial effusion.
**Cameroon**	64	Pericardiocentesis performed immediately in patient with detected pericardial effusion and significant hypotension.
**Cameroon**	65	FAST exam performed within 30 minutes from arrival to the emegency department to exclude hemoperitoneum.
**Cameroon**	66	If GCS <13 at initial assessment or if GCS <15 2 h after injury, then skull X-ray is given.
**Cameroon**	67	Patient arrived with spine immobilized on spinal board or rigid object of similar function

For peer review only

BMJ Open

Perceived usefulness of trauma audit filters in urban India: a mixed-methods multicenter Delphi study comparing filters from the World Health Organization and Low-and-Middle-Income Countries

Journal:	*BMJ Open*
Manuscript ID	bmjopen-2021-059948.R1
Article Type:	Original research
Date Submitted by the Author:	29-Mar-2022

Complete List of Authors:	Berg, Johanna; Karolinska Institutet, Department of Global Public Health; Skånes universitetssjukhus Malmö, Department of Emergency and Internal medicine<br>Alvesson, Helle; Karolinska Institutet, Department of Global Public Health<br>Roy, Nobhojit; The George Institute for Global Health India; Karolinska Institutet, Department of Global Public Health<br>Ekelund, Ulf; Lund University Faculty of Medicine, Department of Clinical Sciences Lund, Emergency Medicine<br>Bains, Lovenish; Maulana Azad Medical College, Department of Surgery; WHO Collaboration Centre for Research in Surgical care Delivery In Low and Middle Income Countries<br>Chatterjee, Shamita; Seth Sukhlal Karnani Memorial Hospital, Department of Surgery<br>Bhattacharjee, Prosanta; RG Kar Medical College, Department of Surgery<br>Siddarth, David; Karolinska Institutet, Department of Global Public Health; Doctors for you<br>Gupta, Swati; Maulana Azad Medical College, Department of Radiodiagnosis and Imaging<br>Kamble, Jyoti; Tata Institute of Social Sciences; Doctors for you<br>Khajanchi, Monty; Seth GS Medical College and KEM Hospital, Department of Surgery; WHO Collaborating Centre for research on surgical care delivery in low and middle income countries<br>Lal, Pawanindra; Maulana Azad Medical College, Department of Surgery<br>Malhotra, Vikas; Maulana Azad Medical College, Department of ENT and Head & Neck Surgery<br>Meher, Ravi; Maulana Azad Medical College, Department of ENT and Head & Neck Surgery<br>Mishra, Anurag; Maulana Azad Medical College, Department of Surgery<br>Mohan, L N; Vydehi Institute of Medical Sciences and Research Centre, Surgery<br>Petzold, Max; University of Gothenburg Sahlgrenska Academy, Public Health and Community Medicine<br>Saxena, Ritu; Lok Nayak Hospital, Department of Accident & Emergency<br>Shrivastava, Prabhat; Maulana Azad Medical College, Department of Burns and Plastic Surgery<br>Singh, Rajdeep; Maulana Azad Medical College, Department of Surgery<br>Soni, Kapil; All India Institute of Medical Sciences, Department of Critical

	and Intensive Care<br>Sural, Sumit; Maulana Azad Medical College, Department of Orthopaedic Surgery<br>Gerdin Wärnberg, Martin ; Karolinska Institutet, Department of Global Public Health; Karolinska University Hospital, Function Perioperative Medicine and Intensive Care
**Primary Subject Heading**:	Emergency medicine
Secondary Subject Heading:	Anaesthesia, Global health, Surgery
Keywords:	ACCIDENT & EMERGENCY MEDICINE, TRAUMA MANAGEMENT, Trauma management < ORTHOPAEDIC & TRAUMA SURGERY

For peer review only - http://bmjopen.bmj.com/site/about/guidelines.xhtml

I, the Submitting Author has the right to grant and does grant on behalf of all authors of the Work (as defined in the below author licence), an exclusive licence and/or a non-exclusive licence for contributions from authors who are: i) UK Crown employees; ii) where BMJ has agreed a CC-BY licence shall apply, and/or iii) in accordance with the terms applicable for US Federal Government officers or employees acting as part of their official duties; on a worldwide, perpetual, irrevocable, royalty-free basis to BMJ Publishing Group Ltd ("BMJ") its licensees and where the relevant Journal is co-owned by BMJ to the co-owners of the Journal, to publish the Work in this journal and any other BMJ products and to exploit all rights, as set out in our licence.

The Submitting Author accepts and understands that any supply made under these terms is made by BMJ to the Submitting Author unless you are acting as an employee on behalf of your employer or a postgraduate student of an affiliated institution which is paying any applicable article publishing charge ("APC") for Open Access articles. Where the Submitting Author wishes to make the Work available on an Open Access basis (and intends to pay the relevant APC), the terms of reuse of such Open Access shall be governed by a Creative Commons licence – details of these licences and which Creative Commons licence will apply to this Work are set out in our licence referred to above.

Other than as permitted in any relevant BMJ Author's Self Archiving Policies, I confirm this Work has not been accepted for publication elsewhere, is not being considered for publication elsewhere and does not duplicate material already published. I confirm all authors consent to publication of this Work and authorise the granting of this licence.

For peer review only

Perceived usefulness of trauma audit filters in urban India: a mixed-methods multicenter Delphi study comparing filters from the World Health Organization and Low-and-Middle-Income Countries

Date: 20 March, 2022

For peer review only

Title page

Word count: 3977

Corresponding author

Johanna Berg (JB)
Karolinska Institutet, Department of Global Public Health; Skåne University hospital, Emergency medicine, department of Emergency and Internal medicine.

e-mail: johanna.berg@ki.se

Postal address: Skånes Universitetssjukhus Malmö, Ruth Lundskogs Gata 5, 214 28, Malmö, SWEDEN

Authors and affiliations

Johanna Berg (JB), MD. Karolinska Institutet, Department of Global Public Health; Skåne University hospital, Emergency medicine, department of Emergency and Internal medicine.

Helle Mölsted Alvesson (HMA), PhD. Karolinska Institutet, Department of Global Public Health; Karolinska Institutet

Nobhojit Roy (NR) MD, MPH, PhD 1.The George Institute for Global Health, New Delhi, INDIA 2. School of Public Health & Preventive Medicine, Monash University, Melbourne, Vic, Australia 3. GPH Karolinska

Ulf Ekelund (UE), MD, PhD. Department of Clinical Sciences Lund, Emergency Medicine, Faculty of Medicine, Lund University, Lund, Sweden

Lovenish Bains (LB), MS Surgery, FNB, FACS, FRCS(Glas) FIAGES, FAIS. Department of Surgery, Maulana Azad Medical College & associated Lok Nayak Hospital, New Delhi, India and Consultant, WHO Collaborating Centre for Research in Surgical Care Delivery in LMICs, India

Shamita Chatterjee (SC), MS, FMAS, FAIS. Professor, Department of Surgery, IPGME&R - Seth Sukhlal Karnani Memorial Hospital, Kolkata, India.

Prosanta Kumar Bhattacharjee (PKB), MS (Surgery), FMAS. Professor of Surgery, R G Kar Medical College, Kolkata. Formally Professor of Surgery, I P G M E & R / S S K M Hospital, Kolkata, India.

Siddarth David (SD), MSc, Disaster Management, Project Manager, Doctors For You, India and Department of Global Public Health, Karolinska Institute, Sweden.

Swati Gupta (SG), MD Radiology, DNB radiology, FRCR, Department of Radiodiagnosis and Imaging, Maulana Azad Medical College & associated Lok Nayak Hospital, New Delhi, India.

Jyoti Kamble (JK), Sr. Research Officer, M.Sc (Biochemistry), Doctors For You, Mumbai, and Department of Public Health, Tata Institute of Social Sciences, Mumbai, India.

Monty Khajanchi (MK), DNB Surgery, Assistant Professor Surgery at Seth GSMC and KEM Hospital, Mumbai. Consultant WHO Collaboration Centre for Research in Surgical Delivery in Low and Middle Income Countries

Pawanindra Lal (PL), MS, DNB, FRCS(Ed,Eng,Glasg, Irel), FACS,FAMS. Director Professor, Department of Surgery, Maulana Azad Medical College & Associated Lok Nayak Hospital,New Delhi, India.

Vikas Malhotra (VM), MS ENT, DNB ENT, FRCS(Glasg), MBA, Department of ENT and Head & Neck Surgery, Maulana Azad Medical College & associated Lok Nayak Hospital, New Delhi, India.

Ravi Meher (RM), MS ENT, DNB ENT, FACS, MNAMS, Department of ENT and Head & Neck Surgery, Maulana Azad Medical College & associated Lok Nayak Hospital, New Delhi, India.

Anurag Mishra (AM), MS, DNB, MNAMS, FAIS, FACS. Department of Surgery, Maulana Azad Medical College & associated Lok Nayak Hospital, New Delhi, India.

L N Mohan (LNM), MBBS, MS, FRCS. Professor of Surgery, Vydehi Institute of Medical Sciences and Research Centre, Bangalore, India

Max Petzold (MP), School of Public Health and Community Medicine, Institute of Medicine, University of Gothenburg, Gothenburg, Sweden

Ritu Saxena (RS), MBBS, MBA (DM), AFIH, Dy. Medical Superintendent, Department of Accident & Emergency, Lok Nayak Hospital, New Delhi, India

Prabhat Shrivastava (PS), MBBS, MS (Surgery), MCh (Plastic Surgery), FICS, FAIS, Consultant and Head, Department of Burns and Plastic Surgery, Lok Nayak Hospital and associated Maulana Azad Medical College, New Delhi, India.

Rajdeep Singh (RS), MS (Surgery), DNB, FRCS (Glasg), FACS. Department of Surgery, Maulana Azad Medical College & associated Lok Nayak Hospital, New Delhi, India.

For peer review only

Kapil Dev Soni (KDS),MD, Critical and Intensive Care,JPN Apex Trauma Center, All India Institute of Medical Sciences, New Delhi, India

Sumit Sural (SS), MS(Ortho.).Department of Orthopaedic Surgery, Maulana Azad Medical College & associated Lok Nayak Hospital, New Delhi, India

Martin Gerdin Wärnberg (MGW), MD, PhD. Karolinska Institutet, Department of Global Public Health; Karolinska University Hospital, Function Perioperative Medicine and Intensive Care

For peer review only

Abstract

Objective: To compare experts' perceived usefulness of audit filters from Ghana, Cameroon, WHO and those locally developed, generate context-appropriate audit filters for trauma care in selected hospitals in urban India and explore characteristics of audit filters that correlate to perceived usefulness. **Design:** A mixed-methods approach using a multi-center online Delphi technique. **Setting:** Two large tertiary hospitals in urban India. **Methods:** Filters were rated on a scale from 1-10 in terms of perceived usefulness, with the option to add new filters and comments. The filters were categorised into three groups depending on their origin; Low-and-Middle-Income Countries (LMIC), World Health Organization (WHO) and New (Locally developed) and their scores compared. Significance was determined using Kruskal–Wallis followed by Wilcox-rank-sum tests. We performed a content analysis of the comments. **Results:** 26 predefined and 15 new filter suggestions were evaluated. The filters had high usefulness scores (mean overall score 9.01 of 10), with the LMIC filters having significantly higher scores compared to those from WHO and those newly added. Three themes were identified in the content analysis relating to *medical relevance*, *feasibility* and *specificity*. **Conclusions:** Audit filters from other LMIC were deemed highly useful in the urban India context. This may indicate that the transferability of defined trauma audit filters between similar contexts is high and that these can provide a starting point when implemented as part of trauma quality improvement programs in low-resource settings.

Keywords: trauma care, trauma, injury, trauma quality improvement, trauma system, audit filter, trauma audit filter, trauma quality indicator

For peer review only

Article summary

• This study is the first to evaluate the usefulness of trauma audit filters from different sources in a low-resource context.

• We used two separate study sites to perform parallel independent online Delphi surveys to compare the usefulness of audit filters for trauma care.

• Both sites scored and selected filters with the intent to implement them.

• The content analysis gave a deeper understanding of the characteristics of the audit filters.

• Due to the nature of the Delphi methodology, the statistical analysis is based on a convenience sample.

For peer review only

Introduction

Trauma, defined as physical injury and the body's associated response, accounts for 4,4 million deaths globally every year.[1] With nearly 90% of these occurring in Low-and-Middle Income Countries (LMIC), it has been estimated that over 2 million lives each year could be saved if fatality rates were reduced to the levels of High-Income Countries (HIC).[2] Strengthening trauma care is essential to the global health agenda and there is evidence that implementing trauma quality improvement programs decreases mortality and improves trauma care in both HIC and LMIC.[3–5] These programs are diverse and involve different approaches to improving care, one of which is audit filters.

Audit filters, in some cases also referred to as quality indicators, are predefined statements that define deviations from standard care. For example, an audit filter could state, *"Patient with Glasgow Coma Scale score <8 should have an endotracheal tube or surgical airway performed before leaving resuscitation area."* A case violating this filter would indicate sub-optimal care and the case flagged for review. Even though there is evidence that TQIP improves outcomes, high-quality evidence on the effects of trauma audit filters, is lacking.[6] The use of audit filters is one technique promoted in the World Health Organization (WHO) guidelines for trauma quality improvement programs.[7] Their implementation requires more extensive resources, compared to other proposed interventions, since they are data-driven.[7]

Audit filters need to be adapted to the context in which they are to be applied.[7,8] To generate context-relevant audit filters, previous studies have used the Delphi technique to identify appropriate filters from a group of experts.[9,10] There is limited experience evaluating the filters proposed by the WHO and their perceived usefulness in a low-resource setting.

India accounts for 20-25% of trauma mortality globally.[1] In-hospital mortality remains high, with one study reporting 30-day mortality of 21%, which is twice as high compared to data from registries in HIC.[11] More than 50% of these deaths have been reported to be preventable.[12] Identifying evidence-based strategies to improve in-hospital trauma care in India is vital.

With this background, this study aims to compare experts' perceived usefulness of audit filters from Ghana, Cameroon, WHO and those locally developed, generate context-appropriate audit filters for trauma care in selected hospitals in urban India and explore characteristics of audit filters that correlate to perceived usefulness.

Material and Methods

Patient and public involvement

We did not involve patients or the public in this study.

For peer review only

Design

This study was nested in the Trauma Audit Filter Trial (TAFT) (ClinicalTrials.gov, ID NCT03235388), which investigates the effect of audit filter implementation on mortality and morbidity in adult trauma patients in urban India.

We conducted a mixed-methods study based on a Delphi technique to facilitate consensus within a panel of experts. [13] The Delphi technique is based on structured, iterative, anonymous surveys where participants can rate and comment on statements. Between each iteration, researchers may report feedback to the panellists to facilitate the discussion. Definition of when consensus is reached in a Delphi is predefined but the exact methods used to define this vary between studies.

The Delphi technique has been used in multiple areas of health care research, including selecting trauma audit filters.[9,10] It is considered particularly useful when investigating complex multidisciplinary problems in areas with limited previous research where considerable uncertainties still exist.

This mixed-methods design allowed us to gather quantitative data from the scoring of the audit filters to answer the question of whether perceived usefulness differed based on the original source as well as select the highest scoring filters for implementation.[14] The qualitative data, the written comments deliberated during the Delphi rounds, was used to explore characteristics of the audit filters and their correlation to the usefulness scores.

We performed two independent Delphi surveys at two different hospitals to allow for the audit filters to be selected and modified based on local priorities and capabilities at each hospital. This design also allowed us to compare scores between two independent groups of experts and see if results were reproducible across the two study sites.

Setting

We conducted this study at two public teaching hospitals Kolkata and Delhi, both with populations of more than 15 million: Seth Sukhlal Karnani Memorial Hospital (SSKM) in Kolkata and Maulana Azad Medical College (MAMC) with associated Lok Nayak Hospital in Delhi. Both are public tertiary teaching hospitals that serve as referral hospitals. The study was conducted online between December 2018 and March 2019.

Ethical approvals

Ethical approvals exist as a part of the TAFT project. In Sweden from the Swedish Ethical Review Authority, 2017/930-31/2 dated 2017-06-07. At MAMC - F.1/IEC/MAMC/(57/02/2017/No113) dated 2017-07-19 and SSKM - IPGME&R/IEC/2017/396 dated 2017-08-21.

Identification of audit filters

We included previously published trauma audit filters intended for use in LMICs. These filters included those from the WHO guidelines[7], as well as filters in one study from

For peer review only

Cameroon[9] and one from Ghana[10]. In total 67 audit filters were identified with 47 from Cameroon[9] and Ghana[10] and 20 from WHO guidelines.[7] We did not deem it feasible to score and comment on all 67 filters at each site and therefore the India based TAFT core research team conducted an internal online Delphi survey to remove duplicate filters, prioritise filters potentially appropriate for the urban setting in India and evaluate the online tool used for this study. The core research team consisted of five participants, four surgeons and one critical care/anaesthesia physician. All participants were at the Professor or Associate professor level and had extensive experience of clinical trauma work and research in urban India. The online Delphi for the core research team was performed in November 2018. The survey consisted of three rounds and response rates were 100% at all rounds. This resulted in 26 selected filters, 5 from WHO and 21 from LMIC sources. (Supplemental material: Table 1)

Participants

We purposefully sampled physicians, surgeons, nurses, and administrators with >5 years of professional experience and involvement in trauma care at the respective hospitals. This was facilitated through the ongoing TAFT project, where local principal investigators with intimate knowledge of the sites could identify experts for inclusion. We chose this approach since considerable knowledge about the local conditions and organisation, in addition to work experience, would be necessary to evaluate the usefulness of the filters at each site. Because we also allowed the filters to be modified and new filters to be suggested, we deemed 10-15 participants feasible to reach a consensus. Before executing the online surveys, a two-day meeting was arranged at the participating hospitals discussing trauma quality improvement programs and introducing the concept of audit filters to the participants.

The online surveys, consensus and stability criteria

We used the online survey software LimeSurvey, to create an anonymous survey for each site.[15] Potential participants were invited to a website with information about the project, where they could register and then take part in the survey.

The audit filters were presented to the participants without revealing their sources. Rating of the perceived usefulness, defined as usefulness from the perspective of experts, was done on an ordinal Likert scale with grades from 1-10. A score of 1 represented not useful and 10 very useful. Open comments could be entered for each filter, and new filters could be proposed. Modifications to the filters could also be suggested. Comments were reviewed and summarised by members of the core research team after completion of each round; these were presented along with the median score of the filter in subsequent rounds.

Filters that received a median score of 7 or higher and newly added filters were included in subsequent rounds. Filters with a score of <7 were immediately rejected. A filter was deemed complete when it was either rejected or reached consensus for the selection, defined as having a median score of 9 or higher over two rounds, or when stable, defined as no significant changes in responses between rounds. A stable filter with a score of <9 was rejected. Stability was determined using the Wilcoxon-rank-sum test, comparing scores

For peer review only

from the previous and current rounds for each filter. A significance level of 0.05 was used. Filters modified based on comments from the panellists were included in subsequent rounds even if they had previously reached a consensus. The iterations continued until all remaining filters reached either consensus or stability. Filters that had a median score of 9 and above were selected for implementation at each site. Reminders were sent via email up to three times if no reply had been recorded.

Statistical analysis of the results

We analysed the sites independently. To compare the perceived usefulness between different sources of audit filters, the filters were categorised into three groups based on their origin (LMIC, WHO, and New). The filters defined in Cameroon and Ghana were combined as the group LMIC, the WHO guidelines as WHO, and new filters proposed by the panellists at the sites as New. Filters that were modified were still included in the group of their origin.

The score from all participants in the final round for each filter was used for analysis. For example, if a filter was rejected in round 2, the scores from all participants in that round were used for analysis. If a filter was selected in round 3, scores from that round and filter were used. We used the Kruskal-Wallis test to compare usefulness scores across the three groups. If this resulted in a significant difference ($p<0.05$), post hoc testing was conducted using the Wilcoxon-rank-sum test to identify differences between the individual groups using a significance level of $p<0.05$. We used the Bonferroni method to adjust p-values for multiple tests. We used R for all analyses.[16]

Content analysis

We explored the characteristics of the audit filters that were discussed using inductive content analysis.[17] We chose this approach as there are limited studies that have described characteristics of audit filters. The team of authors represent a multi-disciplinary group of clinical, social science and epidemiological expertise. Several are physicians and surgeons at participating hospitals.

We collated the comments from both sites. For the initial analysis, the comments were organised so that only the comments and not the associated filters were visible. We did this to detach the comments from the filters and focus on the general problems or characteristics discussed. First, the entire collection of comments was read several times. Then meaning units were identified and abstracted to codes. All codes were sorted into categories that were abstracted into themes. After this, we assessed how the themes and categories compared to usefulness scores and the three source groups; WHO, LMIC and New. We did this by defining groups of low-scoring and high-scoring filters, based on their final filter scores. We then selected a theme or high-level category and assessed which filters and filter groups the theme was associated with. We used frequency analysis to see if the theme may be dominant in any of the specified groups. If this was the case, we returned to the comments associated with this theme and group to understand the specific discussion, from this perspective, better. We used this frequency analysis to help find patterns in the comments that would have been difficult to detect. We developed an audit

For peer review only

trail as the coding was discussed between authors JB, HMA and MGW during the process of analysis. We used the NVivo software for analysis.[18]

Results

Participants

A total of 27 individuals were invited; of these 25 agreed to participate. Participation remained high at both sites throughout rounds (mean participation/round 84%). The distribution of the professions differed across sites, with one site being represented by surgeons and physicians only. (Table: 1).

Table 1: Characteristics of Delphi panellists, by site

Characteristic	Site 1, N = 10[1]	Site 2, N = 15[1]
**Profession**		
Administrator	1 (10%)	0 (0%)
Nurse	2 (20%)	0 (0%)
Physician	1 (10%)	5 (33%)
Surgeon	6 (60%)	10 (67%)
**Department**		
Accident and Emergency	1 (10%)	2 (13%)
Anaesthesia	1 (10%)	2 (13%)
Burns and Plastic Surgery	0 (0%)	1 (6.7%)
Critical care	1 (10%)	0 (0%)
Hospital administration	1 (10%)	0 (0%)
Orthopedic surgery	1 (10%)	2 (13%)
Radiology	0 (0%)	1 (6.7%)
Surgery	5 (50%)	7 (47%)
**Female/Male ratio**		
Female	4 (40%)	3 (20%)
Male	6 (60%)	12 (80%)

[1]n (%)

Surveys

The online surveys were performed from December 2018-March 2019. A total of four rounds were done at each site. The response rates remained stable with high participation rates for each round (Site one: 80-100%, Site two: 93%).

Perceived usefulness

The LMIC filters had the highest mean usefulness score at both sites *(Site one: 9.132, site two; 9.568 of 10)*. Comparing all three source groups; WHO, LMIC and New, we found significant differences in usefulness scores at both sites *(Kruskal-Wallis test: Site one p-value<0.001, site two p-value<0.001).* Comparing pairwise between groups at each site, we found significant differences in usefulness scores between all groups, except between New and WHO filters at site one. *(p-value=1)*. (Fig: 1) Filters that were modified during rounds had no significant difference in scores compared to those that were not modified. *(Site one: p=0.322. Site two: p=0.067.)* (Table 2)

For peer review only

Selection of audit filters

In total, 30 filters for site one and 37 filters for site two were processed, resulting in a selection of 24 and 32 filters, respectively. (Fig: 2)

Table 2: All processed audit filters, source group and final scores at both sites. (Rejected filters highlighted)

Group[a]	ID	Audit filter	Score[b]	Site	Modified[c]
WHO	3.1	Hourly GCS in the emergency department of trauma patients with a diagnosis of skull fracture, intracranial injury or spinal cord injury	10.0	1	Yes
	3.2	Sequential (every 30 minutes) GCS of trauma patients with a diagnosis of skull fracture, intracranial injury or spinal cord injury	10.0	2	Yes
	5	Documentation of history and physical examination by a doctor	10.0	1	No
			10.0	2	No
	6.1	Head computerized tomography (CT) scan done within 2 hours of arrival at hospital for a non-transferred patient with Glasgow Coma Scale score <13 and systolic blood pressure > 90	6.5	1	No
	6.2	Head computerized tomography (CT) scan done within 2 hours of arrival at hospital for a non-transferred patient with Glasgow Coma Scale score < 8 and systolic blood pressure > 90	10.0	2	Yes
	14	Operative treatment of gunshot wound to the abdomen	9.0	1	No
			10.0	2	No
	15.1	Fixation of femoral fracture in adult patient within 24 hours of arrival to emergency department	6.5	1	No
	15.2	Fixation of isolated closed femoral shaft fracture in adult patient within 24 hours of arrival to emergency department	7.0	2	Yes
LMIC	21.1	Vital signs recorded within 5 minutes of arrival to emergency department (must include breathing assessment, heart rate, blood pressure, oxygen saturation if available)	10.0	1	Yes
	21.2	Vital signs recorded within 5 minutes of arrival to emergency department (must include breathing assessment, heart rate, blood pressure, oxygen saturation).	10.0	2	Yes
	22	Senior medical officer made aware of patient with difficulty breathing, or shock present at triage (HR >100, OR SBP <110)* or oxygen saturation <95% within 5 minutes of initial assessment	10.0	1	No
			10.0	2	No
	23	The clinician did assess airway patency by asking the patient a question and listening for a response	10.0	1	No
			10.0	2	No
	24.1	Basic airway manoeuvre assistance (i.e. sweep, chin-lift-jaw-thrust, oral or nasal airway, suction) performed for patient with difficulty or obstructed breathing.	10.0	1	No
	24.2	Basic airway manoeuvre assistance (i.e. jaw-thrust, oral or nasal airway, suction, removal of foreign object) performed for patient with difficulty or obstructed breathing.	10.0	2	Yes
	25	Examination for pneumo- hemothorax done by listening to both sides of the chest with a stethoscope within 5 minutes of patient arrival to emergency department	10.0	1	Yes
			10.0	2	Yes
	26	Chest tube placed within 30 min of patient arrival in patient with suspected or confirmed pneumo- or hemothorax and oxygen saturation less than 98%	9.0	1	No
			10.0	2	No
	27.1	Large bore IV was placed within 5 minutes of patient arrival to the emergency department	10.0	1	Yes

<table>
<tr><th>Group[a]</th><th>ID</th><th>Audit filter</th><th>Score[b]</th><th>Site</th><th>Modified[c]</th></tr>
<tr><td rowspan="24"></td><td>27.2</td><td>Large bore IV was placed within 5 min of patient arrival to the emergency department in patients with tachycardia (heart rate > 110) or hypotension (systolic blood pressure < 90)</td><td>10.0</td><td>2</td><td>Yes</td></tr>
<tr><td rowspan="2">28</td><td rowspan="2">Pressure applied to external bleeding at patient arrival to the emergency department, and maintained until definitive control is performed</td><td>10.0</td><td>1</td><td>No</td></tr>
<tr><td>10.0</td><td>2</td><td>No</td></tr>
<tr><td>30.1</td><td>Reduction and/or splinting with analgesia made for a long bone fracture within 2 hours of admission or prior to transfer</td><td>10.0</td><td>1</td><td>No</td></tr>
<tr><td>30.2</td><td>Splinting with analgesia made for a long bone fracture within 30 minutes of admission or prior to transfer</td><td>10.0</td><td>2</td><td>Yes</td></tr>
<tr><td>35.1</td><td>Burn patient did receive 2-4 mL of crystalloid solution per kilogram body weight per percent body surface burn within 24 hours of injury</td><td>10.0</td><td>1</td><td>No</td></tr>
<tr><td>35.2</td><td>Burn patient did receive 4 mL of Ringer's lactate per kilogram body weight per percent body surface burn within 24 hours of injury</td><td>10.0</td><td>2</td><td>Yes</td></tr>
<tr><td>36.1</td><td>Senior attending physician alerted when airway is compromised, usage jaw thrust, chin lift, ORA/NPA, or suction to open airway</td><td>10.0</td><td>1</td><td>No</td></tr>
<tr><td>36.2</td><td>Senior attending physician alerted within 5 minutes of patient arrival to the emergency department when airway is compromised, usage jaw thrust, chin lift, ORA/NPA, or suction to open airway</td><td>10.0</td><td>2</td><td>Yes</td></tr>
<tr><td rowspan="2">37</td><td rowspan="2">Assessment of mouth/throat for foreign bodies and debris made in patient that has difficulty breathing, within 10 minutes of arrival to emergency department</td><td>10.0</td><td>1</td><td>No</td></tr>
<tr><td>10.0</td><td>2</td><td>No</td></tr>
<tr><td>38.1</td><td>Breathing assessment made within 15 min of arrival to emergency department</td><td>10.0</td><td>1</td><td>No</td></tr>
<tr><td>38.2</td><td>Breathing assessment made within 5 min of arrival to emergency department</td><td>10.0</td><td>2</td><td>Yes</td></tr>
<tr><td>40.1</td><td>Patient assessed for hypovolemia when presenting with hypotension and tachycardia or suspected intra-abdominal bleeding, femoral shaft fracture, or pelvic fracture.</td><td>10.0</td><td>1</td><td>No</td></tr>
<tr><td>40.2</td><td>Patient assessed for hypovolemia using clinical examination, USG, FAST, or DPL within 15 minutes of arrival to the emergency department when presenting with hypotension and tachycardia or suspected intra-abdominal bleeding, femoral shaft fracture, or pelvic fracture.</td><td>10.0</td><td>2</td><td>Yes</td></tr>
<tr><td>45.1</td><td>Laparotomy done within 1 hour of arrival to the emergency department in a patient with abdominal injuries and systolic blood pressure <90</td><td>5.5</td><td>1</td><td>No</td></tr>
<tr><td>45.2</td><td>Laparotomy done within 1 hour of arrival to the emergency department in a patient with abdominal injuries and systolic blood pressure <90 after fluid resuscitation</td><td>10.0</td><td>2</td><td>Yes</td></tr>
<tr><td>46.1</td><td>Immobilization and imaging performed in a patient with suspected spine injury, within 4 hours of arrival to the emergency department</td><td>10.0</td><td>1</td><td>No</td></tr>
<tr><td>46.2</td><td>Immobilization within 10 minutes and imaging performed within 4 hours of arrival to the emergency department in a patient with suspected spine injury</td><td>10.0</td><td>2</td><td>Yes</td></tr>
<tr><td rowspan="2">47</td><td rowspan="2">I.v antibiotics given within 1 hour of arrival to the emergency department in a patient with an open fracture</td><td>10.0</td><td>1</td><td>No</td></tr>
<tr><td>10.0</td><td>2</td><td>No</td></tr>
<tr><td>49.1</td><td>Operation for irrigation and debridement within 12 hours from arrival to emergency department for an open fracture</td><td>10.0</td><td>1</td><td>No</td></tr>
<tr><td>49.2</td><td>Operation for irrigation and debridement within 6 hours from arrival to emergency department in a hemodynamically stable patient with an open fracture</td><td>10.0</td><td>2</td><td>Yes</td></tr>
<tr><td>58.1</td><td>Intubation performed in patient with a GCS score of 8 or less within 30 minutes of arrival to emergency department.</td><td>10.0</td><td>1</td><td>No</td></tr>
</table>

Group[a]	ID	Audit filter	Score[b]	Site	Modified[c]
	58.2	Intubation performed in patient with a GCS score of 8 or less within 10 minutes of arrival to emergency department.	10.0	2	Yes
	62	Operation for sub or epidural hematoma within 3 hours of arrival to emergency department	8.0	1	No
			9.0	2	No
	65	FAST exam performed within 30 minutes from arrival to the emegency department to exclude hemoperitoneum.	10.0	1	No
			9.0	2	No
New	66.1	Antibiotics used in acute major (50% or more) burns within 24 hrs	6.0	1	NA
	66.2	Response time of respective department in attending the call	9.0	2	NA
	67.1	MESS or WHO Trauma scale used in prognosis mangled upper extremity	9.0	1	NA
	67.2	Sample sent for investigations	8.0	2	NA
	68.1	Facial 3 D scan done to rule out facial fractures within 24 hours of arrival to the emergency department	5.0	1	NA
	68.2	Airway breathing and circulation assessed immediately on arrival of patient to the emergency department	10.0	2	NA
	69.1	Serial assessment of vitals and GCS after admission	10.0	1	NA
	69.2	Oxygen therapy with simple face mask initiated in a patient whose SpO2 is less than 92% within 5 minutes of initial assessment of the patient	10.0	2	NA
	70	Response time in initiating definite treatment from arrival to the emergency department, by specialist department, within 1 hour from arrival to the emergency department	9.0	2	NA
	71	Blood components started within 4 hours of arrival to the emergency department if the patient has a Hb < 7g/dl.	9.0	2	NA
	72	AVPU for initial assessment and followed by sequential GCS	9.0	2	NA
	73	Response time of surgeons	8.0	2	NA
	74	Ionotropes in patient of shock	6.0	2	NA
	75	Drug assisted intubation	6.5	2	NA
	76	Sample sent for blood group and cross match in patients with significant bleeding (heart rate > 110 or systolic blood pressure < 90) within 15 minutes of arrival to the emergency department	10.0	2	NA

[a]*Original filter source group.;* [b]*Final median score.;* [c]*Indicates weather the filter was modified by the panellists.*

Characteristics of audit filters in relation to their usefulness

During the content analysis, three major themes relating to an audit filter's feasibility, medical relevance and specificity evolved. Creating audit filters appears to be a balance between medical relevance and feasibility as well as generality and specificity. Logistic limitations may force providers to modify standards of care to make something feasible. For example, if there is limited availability to an operation theatre (OT), someone needs to prioritise which patient should get access first. Defining this in audit filters is a balance between medical relevance (the most injured patient goes to OT first) and feasibility (Limited to one OT, it might not be feasible to state that non-life-threatening injuries should have surgery within 6 hours). The filters need to be general enough to be valid for a substantial part of the patient population but specific enough to state what interventions

need to be done, when they are to be done, and which patients. All themes and high-level subcategories are in table 3.

Table 3: Identified themes during content analysis of comments.

Main themes and subcategories
**Theme 1 Assessing medical relevance**
Filter deemed medically important
Modifying filters based on a medical rationale
Questions medical relevance or correctness
**Theme 2 Questioning feasibility**
Concern that blame will be on the provider
Filter describes something ideal - but not achievable
Logistic limitations
Lacking equipment
Lacking ICU beds
Lacking operation theatre
Lacking staff
Timeframe not feasible
Modifying filters to make them more achievable
Make the filter more conservative
Limiting interventions only for patients that need it
Task shifting
Everyone on the trauma team can do simple interventions
**Theme 3 Increasing filter specificity**
Argues for higher specificity of the filter
Defining cutoffs
Defining target population
Defining time to intervention
Defining wherein the process filter should apply
Defining who should do what

Assessing medical relevance

Filters perceived as medically relevant received high usefulness scores, and filters not perceived as medically relevant received lower scores. This may indicate that one of the main characteristics of an audit filter, for it to be perceived as useful, is that it is perceived as medically relevant. If considered relevant the filters could score highly, even if they had difficult feasibility problems. Filters that originated from WHO were to a higher extent discussed as being less medically relevant than filters from LMIC sources.

For example, discussions on filter 65 **"FAST exam performed within 30 minutes from arrival to the emergency department to exclude hemoperitoneum."**

"Subject to availability (remark: of the ultrasound machine), may be dropped at the moment…"

"FAST should stay as an audit filter as it's integral."

"Don't attempt to reframe this extremely useful filter. Instead channel energies to get FAST for emergency."

In the above example, there is a difficult feasibility problem, the lack of ultrasound machines. However, there was agreement that this intervention is so medically relevant that the filter should be implemented anyway and be used to advocate for ultrasound resources in the emergency department.

Feasibility

In some instances, participants agreed that filters described something medically ideal but not deemed feasible. Reasons for this were lack of resources such as equipment or staff or feasibility in data collection.

For example, while discussing filter 62, **"Operation for sub or epidural hematoma within 3 hours of arrival to emergency department",** panellists commented:

"Feasibility of performing emergency operation within the desired time frame of 3 hours of arrival for this condition remains an issue."

On occasion, if the filter was deemed medically important, it was modified to make it more achievable. This could be done by restricting the patient population that should receive the intervention or prolonging the time to intervention while balancing this towards the medical relevance of the intervention. For example, filters that describe simple airway manoeuvres should be done immediately, whereas a filter stating time to CT may be prolonged.

For example, filter 6 was originally **"Head computerized tomography (CT) scan done within 2 hours of arrival at hospital for a non-transferred patient with Glasgow Coma Scale score <13 and systolic blood pressure > 90".** At one site this was modified from a cutoff of GCS < 13 to GCS < 8 causing a significant change in the target population, increasing the likelihood of significant pathology and reducing the intended use of CT. After this, the filter received high scores and reached a consensus.

Filter specificity

The discussions within this theme reflected that filters could have a varying degree of specificity, that is, how precise they were in defining a target population, an intervention and often a time to that intervention. These cutoffs, to define the target population and time to intervention, were heavily discussed. The cutoffs may not be backed by a high degree of medical evidence and hence become based on consensus.

For peer review only

Example, filter 27: **"Large bore IV was placed within 5 min of patient arrival to the emergency department in patients with tachycardia (heart rate > 110) or hypotension (systolic blood pressure < 90)"**
This filter was agreed upon after four iterations. It contains several cutoffs, one that defines a time to intervention (within 5 minutes), and two thresholds defining the target population should receive the intervention (Patients with HR > 110 or SBP < 90).

This theme intersects with the theme of making filters feasible for implementation because making a filter more conservative may limit the number of patients exposed to an intervention or allow for more time to intervention, making the filter feasible in this context. However, agreeing on specific limits and framing to increase the specificity of the filter was not always done to make it more feasible.

Filter specificity was heavily discussed for filters with high and low usefulness scores within all groups, indicating that specificity is a general characteristic of an audit filter and may not be what deems it as being useful.

Discussion

We found that the filters defined in LMIC had significantly higher usefulness scores and were deemed highly medically relevant compared with filters from the WHO. One reason for this may be that the filters suggested by WHO are derived mainly from filters in mature trauma systems in HIC.[7,8] Filters from LMIC may be reflective of trauma systems more similar to the urban India context.

The American College of Surgeons Committee on Trauma (ACSCOT) published the first audit filters for trauma care improvement in 1987. These 12 filters were derived by expert opinion, and since then, they have evolved and been applied in several trauma systems. [8] Over time it has become clear that filters need to be adapted to local environments, and since 2006, ACSCOT does not list specific filters but recommends filter tracking depending on local priorities. The WHO guidelines also emphasise that the filters listed are potential and that their usefulness may depend on local circumstances.[7] Agreeing on national or world wide quality indicators for trauma care is a challenge, and the perception of what indicators are deemed important varies between regions.[19]

The audit filters defined in Cameroon [9] and Ghana [10] were deemed highly useful in the urban Indian setting. The filters from Cameroon were focused on regional referral hospitals, while those defined in Ghana were intended for use in district-level hospitals. Many of these filters represent the initial management, resuscitation and simple, potentially life-saving interventions like airway manouvers or placement of intravenous catheters. These filters may not be perceived as useful in a mature trauma system where these interventions are a part of routine care, firmly built-in by years of improving training, processes and access to equipment.

Several studies from HIC have struggled to determine the effect on outcomes of specific audit filters. The review process itself, being costly and labour-intensive, has raised questions about the usefulness of audit filters in well-developed trauma systems.[8,20]

For peer review only

Their potential use in less developed trauma systems is less studied but seems promising. One study by Chadbunchachai et al. in Thailand showed a significant reduction of preventable trauma deaths between 1994 and 1997 after implementing trauma audit filters.[5,21] The discrepancy that the same filter may be useful and potentially influence outcomes in one context and not the other highlights the importance of audit filters being selected and evolved locally to suit the context of their use.

Hypothetically, applying filters developed in HIC and mature trauma systems to a low-resource setting may be disruptive since they may divert focus from the more pressing concerns and hence not improve the processes that could affect outcomes in that context.

The LMIC definition is broad; we have chosen to use it here as a distinction against HIC with mature trauma systems. Cameroon, Ghana and India would be classified more specifically as lower-middle-income countries. For low-income countries, there is very limited research on trauma systems.[4] Whether the filters evaluated in this study would be transferable to low-income countries is unclear. Treatment of a majority of trauma patients requires a health care infrastructure that can supply surgical services. This is lacking in several contexts, especially in low-income, lower-middle-income countries and in rural areas.[22]

We found that the new suggested filters at both sites had the lowest median scores. This may reflect the difficulty of defining audit filters based on consensus with online survey interaction only. Developing new audit filters that are clear, feasible and specific is a complex task. If this is attempted via an online survey, it may be beneficial to supply the participants with a short suggestion on how an audit filter may be formulated to make sure it is specific, highlights the medical relevance and is feasible in the context.

One of this study's main strengths is the nesting within the TAFT project since both sites performed the surveys to select audit filters for implementation in the hospital. The use of two separate sites adds strength to our quantitative analysis, as both sites showed similar results. The online Delphi approach enabled us to execute a study at two sites in different cities, with a wide range of participants at a very low cost. Similar studies could be replicated in other low-resource settings with standard technical devices.

There are several limitations to our study. Firstly, the pre-selection of audit filters made by the core team might have induced bias and contributed to the overall high usefulness scores of the filters. However, analysis of the results of this pre-selection Delphi did not reveal any significant difference in usefulness scores between filters from WHO and LMIC during the pre-selection. (Supplemental material: Figure 1) Secondly, the number of filters from the WHO was lower, and one filter receiving low scores had a large impact on the mean for this group. Thirdly, the statistical analysis is based on a convenience sample. The inclusion of participants does not represent a randomised sample, and the overall high scores contribute to a ceiling effect which may affect the results of the statistical analysis. Fourthly, the generalisability of the selected filters is limited because of the local modifications to the filters, based on a smaller number of experts at each site. Lastly, the content analysis was done based on comments, not by directed questions. It was not mandatory to leave a comment, and hence this discussion primarily focuses on problems or

For peer review only

features to improve. Because the main focus of the discussion was to agree on something standardised and measurable, very little about experiences or deeper mechanisms can be extracted from this material. We believe, however, that the content analysis gives another level of understanding as to why filters received the scores that they did, which may help guide efforts when adapting audit filters in other contexts.

We also want to highlight that our expert panels mainly consisted of surgeons and physicians, and perspectives from other health care personnel were limited. Management of the trauma patient involves a broad team of personnel and including panellists who reflect this is important for future studies in this area.

Conclusion

The trauma audit filters defined in Ghana and Cameroon were deemed highly useful in the urban India context, indicating that the potential for transferability of trauma audit filters between similar contexts is high. These filters may be used as a starting ground when implementing trauma improvement programs including audit filters in similar settings. Compared to filters derived from high-resource programs, many of these filters focus on initial interventions and resuscitation and were deemed to have high medical relevance in this context.

For peer review only

Acknowledgements

We would like to thank all the panellists in the Delphi survey for their time and dedication to this project. Their constructive work in refining the audit filters is what made this study possible. A warm thank you to the participating centres, where the efforts by a great many continue as these filters are implemented and continuously used for review. Finally, thank you to all the participants in the ongoing Trauma Audit Filter Trial: clinicians, health care staff, project officers and mostly all patients and relatives that generously allow us to include them.

Contributorship statement

MGW, NR, KDS, MK conceived the study as part of the Trauma Audit Filter Trial. JB, MGW, HMA, NR, KDS, MK contributed to the design of the study. MGW, NR, KDS, MK and SD conducted the on-site training before the online Delphi. JB, MGW, NR, LB, PKB, SC, SD, SG, MK, PL, VM, RM, AM, LNM, RS, PS, RS, KDS and SS were a part of the execution of the online Delphi. JB prepared the data. JB and MGW conducted the statistical analysis. MP reviewed the statistical analysis. JB performed the content analysis in collaboration with HMA and MGW. JB and MGW drafted the first version of the article. The interpretation of data and critical revisions of the work for important intellectual content was performed by JB, MGW, HMA, NR, LB, PKB, SC, SD, SG, MK, PL, VM, RM, AM, LNM, RS, PS, RS, KDS, SS, MP, UE and JK.

Competing interests

None declared.

Funding

This work was supported by the Swedish Research Council (2016-02041).

Data sharing statement

Data and R-code for the quantitative analysis are publicly available on GitHub at https://github.com/titco/utaf. To protect the privacy of the participants the qualitative data is not available.

Figure 1: Usefulness scores and level of significance for trauma audit filters by source group

Figure 2: All Delphi iterations and filter selection based on identified filters, by site

For peer review only

References

1 GBD 2017 Causes of Death Collaborators. Global, regional, and national age-sex-specific mortality for 282 causes of death in 195 countries and territories, 1980-2017: a systematic analysis for the Global Burden of Disease Study 2017. *Lancet (London, England)* 2018;**392**:1736–88. doi:10.1016/s0140-6736(18)32203-7

2 Kotagal M, Agarwal-Harding KJ, Mock C, *et al.* Health and Economic Benefits of Improved Injury Prevention and Trauma Care Worldwide. *PLoS ONE* 2014;**9**:e91862. doi:10.1371/journal.pone.0091862

3 Juillard CJ, Mock C, Goosen J, *et al.* Establishing the Evidence Base for Trauma Quality Improvement: A Collaborative WHO-IATSIC Review. *World journal of surgery* 2009;**33**:1075–86. doi:10.1007/s00268-009-9959-8

4 Reynolds TA, Stewart B, Drewett I, *et al.* The Impact of Trauma Care Systems in Low- and Middle-Income Countries. *Annual review of public health* 2017;**38**:507–32. doi:10.1146/annurev-publhealth-032315-021412

5 The International Society of Surgery (ISS) and the G4 Alliance International Standards and Guidelines for Quality Safe Surgery and Anesthesia (ISG-QSSA), Jin J, Akau'ola S, *et al.* Effectiveness of Quality Improvement Processes, Interventions, and Structure in Trauma Systems in Low- and Middle-Income Countries: A Systematic Review and Meta-analysis. *World Journal of Surgery* 2021;1–7. doi:10.1007/s00268-021-06065-9

6 Evans C, Howes D, Pickett W, *et al.* Audit filters for improving processes of care and clinical outcomes in trauma systems. *Cochrane Database of Systematic Reviews* 2009;**84**:782. doi:10.1002/14651858.cd007590.pub2

7 World Health Organization. *Guidelines for trauma quality improvement programmes*. 2009. https://apps.who.int/iris/handle/10665/44061

8 Ronald Maier, Michael Rhodes. Trauma Performance Improvement. In: *Injury control: A guide to research and program evaluation*. Cambridge: : Cambridge University Press 236–49. doi:DOI: 10.1017/CBO9780511530234.017

9 Wu N, Carvalho M, Nwanna-Nzewunwa O, *et al.* Developing Trauma Audit Filters for Regional Referral Hospitals in Cameroon: Mixed-Methods Approach. *The Journal of surgical research* 2018;**232**:578–86. doi:10.1016/j.jss.2018.07.048

10 Stewart BT, Gyedu A, Quansah R, *et al.* District-level hospital trauma care audit filters: Delphi technique for defining context-appropriate indicators for quality improvement initiative evaluation in developing countries. *Injury* 2016;**47**:211–9. doi:10.1016/j.injury.2015.09.007

11 Roy N, Gerdin M, Ghosh S, *et al.* 30-Day In-hospital Trauma Mortality in Four Urban University Hospitals Using an Indian Trauma Registry. *World Journal of Surgery* 2016;**40**:1299–307. doi:10.1007/s00268-016-3452-y

For peer review only

12 Roy N, Veetil DK, Khajanchi MU, *et al.* Learning from 2523 trauma deaths in India-opportunities to prevent in-hospital deaths. *BMC Health Services Research* 2017;**17**:142. doi:10.1186/s12913-017-2085-7

13 Hsu C-C, Sandford BA. The Delphi Technique: Making Sense Of Consensus. *Practical Assessment, Research & Evaluation* 2007;**12**. doi:doi.org/10.7275/pdz9-th90

14 Berg J. Dataset titco/utaf Zenodo. Published Online First: March 2022. doi:10.5281/zenodo.6371221

15 GmbH. LimeSurvey: An Open Source survey tool. 2006.https://www.limesurvey.org/

16 R Foundation for Statistical Computing. R: A language and environment for statistical computing. https://www.r-project.org/

17 Krippendorff K. *Content analysis : an introduction to its methodology*. Fourth edition. SAGE 2018.

18 QSR International. NVivo Qualitative Data Analysis Software. 2020.https://www.qsrinternational.com/nvivo-qualitative-data-analysis-software/home

19 Coccolini F, Kluger Y, Moore EE, *et al.* Trauma quality indicators: internationally approved core factors for trauma management quality evaluation. *World Journal of Emergency Surgery : WJES* 2021;**16**:6. doi:10.1186/s13017-021-00350-7

20 Willis CD, Gabbe BJ, Cameron PA. Measuring quality in trauma care. *Injury* 2007;**38**:527–37. doi:10.1016/j.injury.2006.06.018

21 Chadbunchachai W, Sriwiwat S, Kulleab S, *et al.* The comparative study for quality of trauma treatment before and after the revision of trauma audit filter, Khon Kaen hospital 1998. *Journal of the Medical Association of Thailand = Chotmaihet thangphaet* 2001;**84**:782–90.

22 Meara JG, Leather AJM, Hagander L, *et al.* Global Surgery 2030: evidence and solutions for achieving health, welfare, and economic development. *The Lancet* 2015;**386**:569–624. doi:10.1016/s0140-6736(15)60160-x

For peer review only

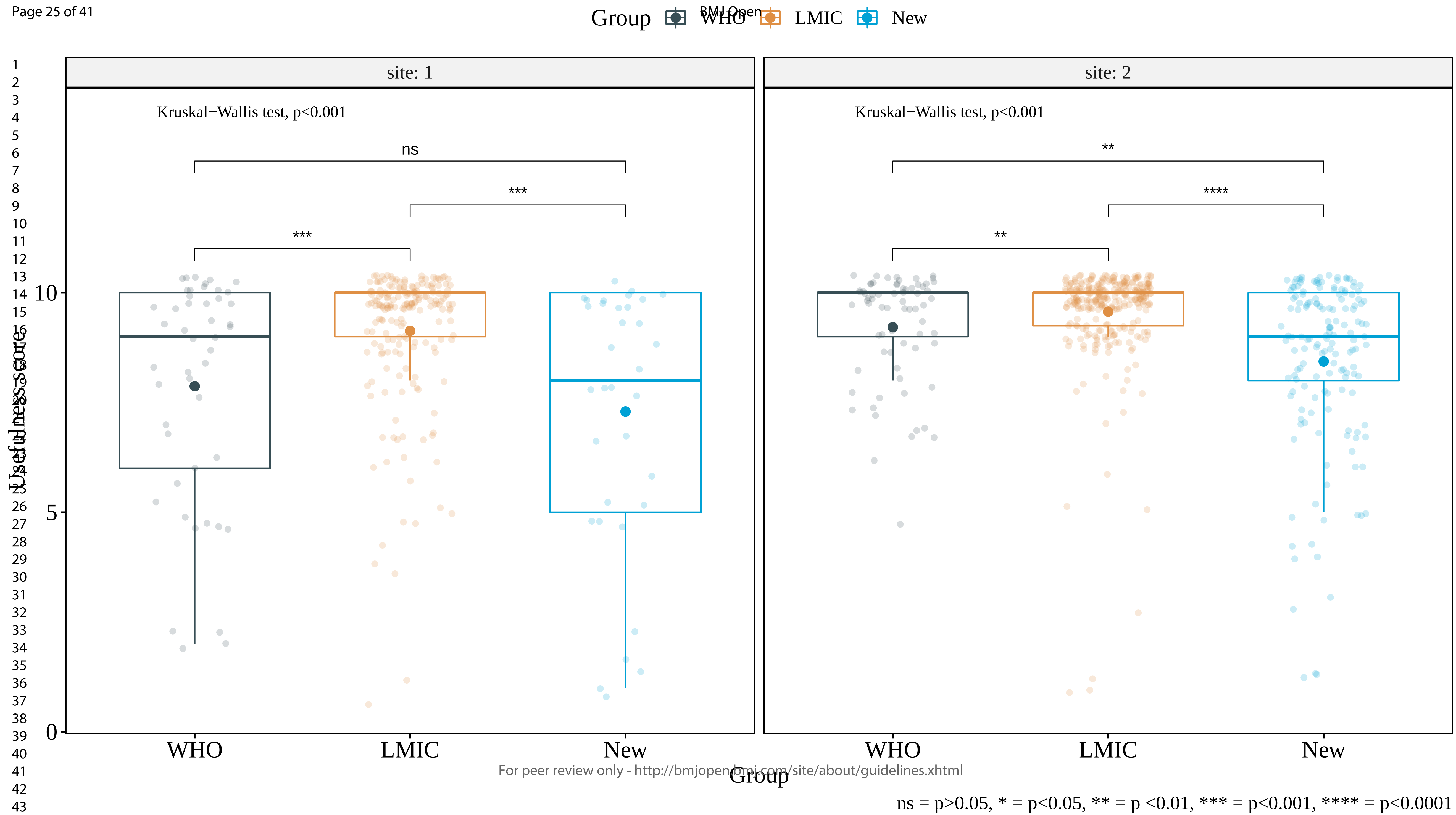
Group WHO LMIC New
site: 1
site: 2
Kruskal−Wallis test, p<0.001
ns
***
***
Kruskal−Wallis test, p<0.001
**
****
**
Usefulness score
10
5
0
WHO
LMIC
New
Group
ns = p>0.05, * = p<0.05, ** = p <0.01, *** = p<0.001, **** = p<0.0001

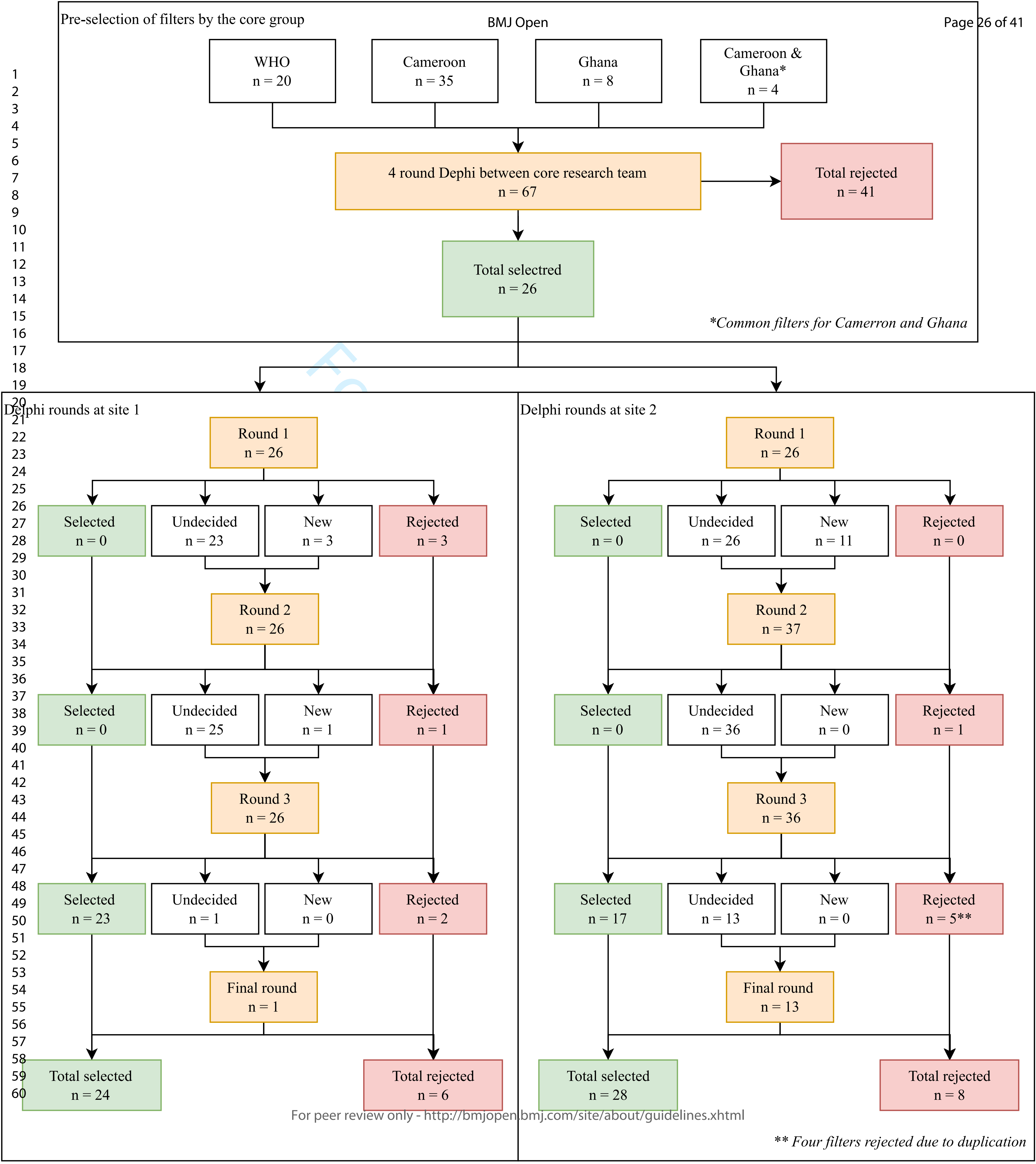
Pre-selection of filters by the core group
WHO n = 20
Cameroon n = 35
Ghana n = 8
Cameroon & Ghana* n = 4
4 round Dephi between core research team n = 67
Total rejected n = 41
Total selectred n = 26
*Common filters for Camerron and Ghana
Delphi rounds at site 1
Round 1 n = 26
Selected n = 0
Undecided n = 23
New n = 3
Rejected n = 3
Round 2 n = 26
Selected n = 0
Undecided n = 25
New n = 1
Rejected n = 1
Round 3 n = 26
Selected n = 23
Undecided n = 1
New n = 0
Rejected n = 2
Final round n = 1
Total selected n = 24
Total rejected n = 6
Delphi rounds at site 2
Round 1 n = 26
Selected n = 0
Undecided n = 26
New n = 11
Rejected n = 0
Round 2 n = 37
Selected n = 0
Undecided n = 36
New n = 0
Rejected n = 1
Round 3 n = 36
Selected n = 17
Undecided n = 13
New n = 0
Rejected n = 5**
Final round n = 13
Total selected n = 28
Total rejected n = 8
** Four filters rejected due to duplication

Supplemental material

01/03/2022

Supplemental material

Analysis of the pre-Delphi by the core team

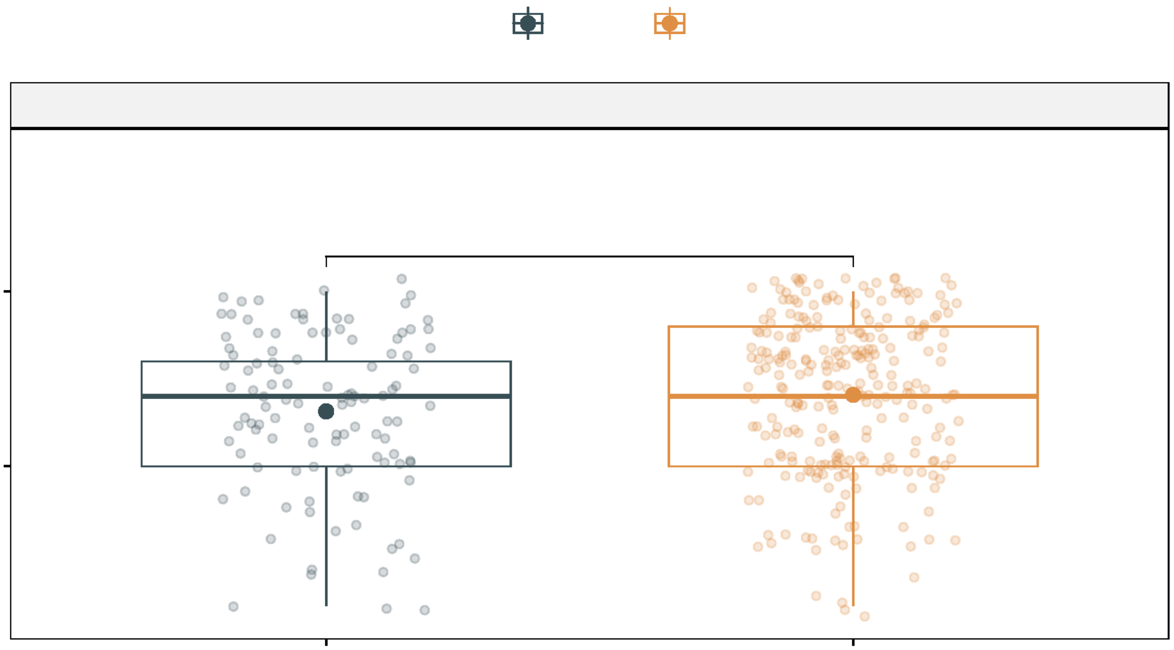

Figure 1: Pre-Delphi: Usefulness scores and level of significance for trauma audit filters by source group

For peer review only - http://bmjopen.bmj.com/site/about/guidelines.xhtml

All initial audit filters

Table 1: All initial audit filters. n = 67

Group	ID	Audit filter
WHO	1	Response time of surgeons
WHO	2	Availability of operating room
WHO	3	Sequential GCS in the emergency department of trauma patients with a diagnosis of skull fracture, intracranial injury or spinal cord injury
WHO	4	At least hourly, determination and recordings of blood pressure, pulse, respirations, temperature, Glasgow Coma Scale (GCS) score and intake and output (I&O) measurements for a major or severe trauma patient done. Beginning with arrival in the resuscitation area and including time spent in radiology up to admission to the operating room or ICU, death, or transfer to another hospital
WHO	5	Documentation of history and physical examination by a doctor
WHO	6	Head computerized tomography (CT) scan done within 2 hours of arrival at hospital for a non-transferred patient with Glasgow Coma Scale score <13
WHO	7	Endotracheal tube or surgical airway performed before leaving resuscitation area in patient with Glasgow Coma Scale score <8
WHO	8	No laparotomy within 1 hour of arrival at the hospital for patient with abdominal injuries and hypotension (systolic BP <90)
WHO	9	Laparotomy not performed (within 4 hours to or 24 hours after admission depending on hospital practice)
WHO	10	Craniotomy performed within 4 hours of arrival to the emergency department, for drainage of epidural or subdural haematoma
WHO	11	Abdominal, thoracic, vascular or cranial surgery after 24 hours
WHO	12	Unplanned return to operating theatre within 48 hours of initial procedure
WHO	13	Patient requiring re-intubation of the airway within 48 hours of extubation
WHO	14	Operative treatment of gunshot wound to the abdomen
WHO	15	Fixation of femoral fracture in adult patient within 24 hours of arrival to emergency department
WHO	16	All delays in identification of injuries
WHO	17	All trauma deaths
WHO	18	Required equipment, shared with other departments (e.g. fluid warmer, ventilator), not immediately available when requested
WHO	19	Non-compliance with institutional protocols
WHO	20	All major complications (e.g. deep venous thrombosis, pulmonary embolus, decubitus ulcers
Ghana and Cameroon	21	Vital signs recorded within 15 minutes of arrival (must include breathing assessment, heart rate, blood pressure, oxygen saturation if available)
Ghana	22	Senior medical officer made aware of patient with difficulty breathing, OR shock present at triage (HR >100, OR SBP <110)* OR oxygen saturation <95% within 5 minutes of initial assessment
Ghana	23	The clinician did assess airway patency by asking the patient a question and listening for a response
Ghana	24	Basic airway manoeuvre assistance (i.e. sweep, chin-lift-jaw-thrust, oral or nasal airway, suction) performed for patient with difficulty or obstructed breathing.
Ghana	25	Examination for pneumo- hemothorax done by listening to both sides of the chest with a stethoscope and bilateral percussion, within 60 minutes of patient arrival to emergency department

For peer review only

Group	ID	Audit filter
**Ghana**	26	Chest tube placed within 30 min of patient arrival in patient with suspected or confirmed pneumo- or hemothorax and oxygen saturation less than 98%
**Ghana**	27	Large bore IV was placed within 15 min of patient arrival to the emergency department
**Ghana and Cameroon**	28	Pressure applied to external bleeding at patient arrival, and maintained until definitive control is performed
**Ghana and Cameroon**	29	If AVPU is not 'A' AND the patient is not in shock, the head of the bed is elevated to 45 degrees.
**Ghana**	30	Reduction and/or splinting with analgesia made for a long bone fracture within 2 h of admission or prior to transfer
**Ghana and Cameroon**	31	Patient is completely undressed, fully examined and covered for privacy within 30 min of arrival
**Ghana**	32	Fluid order for a burn patient not done using the Parkland formula is recorded within 1 h for burns over 15% total body surface area that occurred less than 24 h from patient arrival
**Cameroon**	33	No sufficient history from patient or next best historian obtained.
**Cameroon**	34	No emergency department team prepared and ready with sufficient staff before patient arrival.
**Cameroon**	35	Burn patient did not receive 2-4 mL of crystalloid solution per kilogram body weight per percent body surface burn within 24 h of injury
**Cameroon**	36	Senior attending physician alerted when airway is compromised, usage jaw thrust, chin lift, ORA/NPA, or suction to open airway
**Cameroon**	37	Assessment of mouth/throat for foreign bodies and debris made in patient that has difficulty breathing, within 10 minutes of arrival to emergency department
**Cameroon**	38	Breathing assessment made within 15 min of arrival to emergency department
**Cameroon**	39	Establish two large bore IVs within 15 min of arrival.
**Cameroon**	40	Patient assessed for hypovolemia when presenting with hypotension and tachycardia or suspected intra-abdominal bleeding, femoral shaft fracture, or pelvic fracture.
**Cameroon**	41	Neurological assessment made with AVPU.
**Cameroon**	42	If patient arrived on spinal board, when appropriate patient is subsequently safely logrolled off board.
**Cameroon**	43	Open wounds are treated and covered
**Cameroon**	44	Chest X-ray performed for pneumothorax, hemothorax, and flail chest diagnosis.
**Cameroon**	45	Laparotomy not done within 1 h of arrival to hospital in a patient with abdominal injuries and systolic blood pressure <90
**Cameroon**	46	Immobilization and imaging performed in a patient with suspected spine injury, within 4 hours of arrival to the emergency department
**Cameroon**	47	I.v antibiotics given within 1 hour of arrival to the emergency department in a patient with an open fracture
**Cameroon**	48	Long bone fracture is reduced with analgesia and/or splinted within 4 h of admission or prior to transfer.
**Cameroon**	49	Operation for irrigation and debridement within 12 h from arrival to emergency department for an open fracture
**Cameroon**	50	Before patient arrival, emergency department is adequately stocked with essential supplies.
**Cameroon**	51	Referral made within 15 min of determination if treatment of injury is beyond site's capabilities (i.e. not able to operate).
**Cameroon**	52	Second surgery within 48 h of previous operation

For peer review only

Group	ID	Audit filter
Cameroon	53	Unplanned return to operating theater.
Cameroon	54	Unplanned intubation.
Cameroon	55	Unplanned return to intensive care unit.
Cameroon	56	Unplanned readmission.
Cameroon	57	No ventilation done with bag-valve mask in patient who can not self ventilate.
Cameroon	58	Intubation performed in patient with a GCS score of 8 or less.
Cameroon	59	Thoracostomy within 30 min of arrival to emergency department in patient with pneumothorax or haemothorax
Cameroon	60	Patient body temperature is maintained by giving warmed IV fluids and blood to prevent hypothermia
Cameroon	61	If intracranial pressure <20 mm Hg, patient treated with mannitol or hypertonic saline
Cameroon	62	Operation for sub or epidural hematoma within 3 hours of arrival to emergency department
Cameroon	63	No FAST exam performed to evaluate for pericardial effusion.
Cameroon	64	Pericardiocentesis performed immediately in patient with detected pericardial effusion and significant hypotension.
Cameroon	65	FAST exam performed within 30 minutes from arrival to the emegency department to exclude hemoperitoneum.
Cameroon	66	If GCS <13 at initial assessment or if GCS <15 2 h after injury, then skull X-ray is given.
Cameroon	67	Patient arrived with spine immobilized on spinal board or rigid object of similar function

For peer review only

Welcome to the first round of the Trauma Audit Filters Trial delphi survey to develop locally relevant audit filters!

Your task is to rate each statement on the following pages on a scale from 1 to 10, where 1 represents not useful and 10 very useful. A statement is useful if:

1. Failure to perform the action depicted in the statement is likely to result in poor patient outcome or complications 2. It is feasible to collect data on the statement in your setting.

If you want, you may comment on statements. For example, if you find that the time frame of a statement is not feasible, you may write so in the comment, and suggest a new time frame. At the end of the survey you may suggest new statements, or filters, that you think would be useful to track quality of trauma care at your centre.

Thank you for participating!

Please send an email to Martin Gerdin Wärnberg at martin.gerdin@ki.se if you have any questions.

Section A: Audit filters

A1. **Filter ID: TAFT3Sequential GCS in the emergency department of trauma patients with a diagnosis of skull fracture, intracranial injury or spinal cord injury**

	1 - Not useful	2	3	4	5	6	7	8	9	10 - Very useful
Rate the usefulness of this audit filter	☐	☐	☐	☐	☐	☐	☐	☐	☐	☐

A2. **Add a comment about this audit filter**

A3. **Filter ID: TAFT5Documentation of history and physical examination by a doctor**

	1 - Not useful	2	3	4	5	6	7	8	9	10 - Very useful
Rate the usefulness of this audit filter	☐	☐	☐	☐	☐	☐	☐	☐	☐	☐

A4. **Add a comment about this audit filter**

A5. **Filter ID: TAFT6Head computerized tomography (CT) scan done within 2 hours of arrival at hospital for a non-transferred patient with Glasgow Coma Scale score 90**

	1 - Not useful	2	3	4	5	6	7	8	9	10 - Very useful
Rate the usefulness of this audit filter	☐	☐	☐	☐	☐	☐	☐	☐	☐	☐

A6. **Add a comment about this audit filter**

A7. **Filter ID: TAFT14Operative treatment of gunshot wound to the abdomen**

	1 - Not useful	2	3	4	5	6	7	8	9	10 - Very useful
Rate the usefulness of this audit filter	☐	☐	☐	☐	☐	☐	☐	☐	☐	☐

A8. **Add a comment about this audit filter**

Section B: Audit filters

B1. **Filter ID: TAFT15Fixation of femoral fracture in adult patient within 24 hours of arrival to emergency department**

	1 - Not useful	2	3	4	5	6	7	8	9	10 - Very useful
Rate the usefulness of this audit filter	☐	☐	☐	☐	☐	☐	☐	☐	☐	☐

B2. **Add a comment about this audit filter**

B3. **Filter ID: TAFT21Vital signs recorded within 15 minutes of arrival to emergency department (must include breathing assessment, heart rate, blood pressure, oxygen saturation if available)**

	1 - Not useful	2	3	4	5	6	7	8	9	10 - Very useful
Rate the usefulness of this audit filter	☐	☐	☐	☐	☐	☐	☐	☐	☐	☐

B4. **Add a comment about this audit filter**

B5. **Filter ID: TAFT22Senior medical officer made aware of patient with difficulty breathing, or shock present at triage (HR >100, OR SBP**

	1 - Not useful	2	3	4	5	6	7	8	9	10 - Very useful
Rate the usefulness of this audit filter	☐	☐	☐	☐	☐	☐	☐	☐	☐	☐

B6. **Add a comment about this audit filter**

B7. **Filter ID: TAFT23The clinician did assess airway patency by asking the patient a question and listening for a response**

	1 - Not useful	2	3	4	5	6	7	8	9	10 - Very useful
Rate the usefulness of this audit filter	☐	☐	☐	☐	☐	☐	☐	☐	☐	☐

For peer review only - http://bmjopen.bmj.com/site/about/guidelines.xhtml

B8. **Add a comment about this audit filter**

Section C: Audit filters

C1. **Filter ID: TAFT24Basic airway manoeuvre assistance (i.e. sweep, chin-lift-jaw-thrust, oral or nasal airway, suction) performed for patient with difficulty or obstructed breathing.**

	1 - Not useful	2	3	4	5	6	7	8	9	10 - Very useful
Rate the usefulness of this audit filter	☐	☐	☐	☐	☐	☐	☐	☐	☐	☐

C2. **Add a comment about this audit filter**

C3. **Filter ID: TAFT25Examination for pneumo- hemothorax done by listening to both sides of the chest with a stethoscope within 15 minutes of patient arrival to emergency department**

	1 - Not useful	2	3	4	5	6	7	8	9	10 - Very useful
Rate the usefulness of this audit filter	☐	☐	☐	☐	☐	☐	☐	☐	☐	☐

C4. **Add a comment about this audit filter**

C5. **Filter ID: TAFT26Chest tube placed within 30 min of patient arrival in patient with suspected or confirmed pneumo- or hemothorax and oxygen saturation less than 98%**

	1 - Not useful	2	3	4	5	6	7	8	9	10 - Very useful
Rate the usefulness of this audit filter	☐	☐	☐	☐	☐	☐	☐	☐	☐	☐

C6. **Add a comment about this audit filter**

C7. **Filter ID: TAFT27Large bore IV was placed within 15 min of patient arrival to the emergency department**

	1 - Not useful	2	3	4	5	6	7	8	9	10 - Very useful
Rate the usefulness of this audit filter	☐	☐	☐	☐	☐	☐	☐	☐	☐	☐

C8. **Add a comment about this audit filter**

Section D: Audit filters

D1. **Filter ID: TAFT28Pressure applied to external bleeding at patient arrival to the emergency department, and maintained until definitive control is performed**

	1 - Not useful	2	3	4	5	6	7	8	9	10 - Very useful
Rate the usefulness of this audit filter	☐	☐	☐	☐	☐	☐	☐	☐	☐	☐

D2. **Add a comment about this audit filter**

D3. **Filter ID: TAFT30Reduction and/or splinting with analgesia made for a long bone fracture within 2 hours of admission or prior to transfer**

	1 - Not useful	2	3	4	5	6	7	8	9	10 - Very useful
Rate the usefulness of this audit filter	☐	☐	☐	☐	☐	☐	☐	☐	☐	☐

D4. **Add a comment about this audit filter**

D5. **Filter ID: TAFT35Burn patient did receive 2-4 mL of crystalloid solution per kilogram body weight per percent body surface burn within 24 hours of injury**

	1 - Not useful	2	3	4	5	6	7	8	9	10 - Very useful
Rate the usefulness of this audit filter	☐	☐	☐	☐	☐	☐	☐	☐	☐	☐

D6. **Add a comment about this audit filter**

D7. **Filter ID: TAFT36Senior attending physician alerted when airway is compromised, usage jaw thrust, chin lift, ORA/NPA, or suction to open airway**

	1 - Not useful	2	3	4	5	6	7	8	9	10 - Very useful
Rate the usefulness of this audit filter	☐	☐	☐	☐	☐	☐	☐	☐	☐	☐

D8. **Add a comment about this audit filter**

Section E: Audit filters

E1. **Filter ID: TAFT37Assessment of mouth/throat for foreign bodies and debris made in patient that has difficulty breathing, within 10 minutes of arrival to emergency department**

	1 - Not useful	2	3	4	5	6	7	8	9	10 - Very useful
Rate the usefulness of this audit filter	☐	☐	☐	☐	☐	☐	☐	☐	☐	☐

E2. **Add a comment about this audit filter**

E3. **Filter ID: TAFT38Breathing assessment made within 15 min of arrival to emergency department**

	1 - Not useful	2	3	4	5	6	7	8	9	10 - Very useful
Rate the usefulness of this audit filter	☐	☐	☐	☐	☐	☐	☐	☐	☐	☐

E4. **Add a comment about this audit filter**

E5. **Filter ID: TAFT40Patient assessed for hypovolemia when presenting with hypotension and tachycardia or suspected intra-abdominal bleeding, femoral shaft fracture, or pelvic fracture.**

	1 - Not useful	2	3	4	5	6	7	8	9	10 - Very useful
Rate the usefulness of this audit filter	☐	☐	☐	☐	☐	☐	☐	☐	☐	☐

E6. **Add a comment about this audit filter**

E7. **Filter ID: TAFT45Laparotomy done within 1 hour of arrival to the emergency department in a patient with abdominal injuries and systolic blood pressure**

	1 - Not useful	2	3	4	5	6	7	8	9	10 - Very useful
Rate the usefulness of this audit filter	☐	☐	☐	☐	☐	☐	☐	☐	☐	☐

E8. **Add a comment about this audit filter**

Section F: Audit filters

F1. **Filter ID: TAFT46Immobilization and imaging performed in a patient with suspected spine injury, within 4 hours of arrival to the emergency department**

Rate the usefulness of this audit filter: 1 - Not useful ☐ 2 ☐ 3 ☐ 4 ☐ 5 ☐ 6 ☐ 7 ☐ 8 ☐ 9 ☐ 10 - Very useful ☐

F2. **Add a comment about this audit filter**

F3. **Filter ID: TAFT47I.v antibiotics given within 1 hour of arrival to the emergency department in a patient with an open fracture**

Rate the usefulness of this audit filter: 1 - Not useful ☐ 2 ☐ 3 ☐ 4 ☐ 5 ☐ 6 ☐ 7 ☐ 8 ☐ 9 ☐ 10 - Very useful ☐

F4. **Add a comment about this audit filter**

F5. **Filter ID: TAFT49Operation for irrigation and debridement within 12 hours from arrival to emergency department for an open fracture**

Rate the usefulness of this audit filter: 1 - Not useful ☐ 2 ☐ 3 ☐ 4 ☐ 5 ☐ 6 ☐ 7 ☐ 8 ☐ 9 ☐ 10 - Very useful ☐

F6. **Add a comment about this audit filter**

F7. **Filter ID: TAFT58Intubation performed in patient with a GCS score of 8 or less within 30 minutes of arrival to emergency department.**

Rate the usefulness of this audit filter: 1 - Not useful, 2, 3, 4, 5, 6, 7, 8, 9, 10 - Very useful

F8. **Add a comment about this audit filter**

Section G: Audit filters

G1. **Filter ID: TAFT62Operation for sub or epidural hematoma within 3 hours of arrival to emergency department**

Rate the usefulness of this audit filter: 1 - Not useful, 2, 3, 4, 5, 6, 7, 8, 9, 10 - Very useful

G2. **Add a comment about this audit filter**

G3. **Filter ID: TAFT65FAST exam performed within 30 minutes from arrival to the emegency department to exclude hemoperitoneum.**

Rate the usefulness of this audit filter: 1 - Not useful, 2, 3, 4, 5, 6, 7, 8, 9, 10 - Very useful

G4. **Add a comment about this audit filter**

G5. **If you wish to propose new audit filters, please enter them here.**
Separate your suggested filters with a semicolon, for example
Response time of surgeons; Neurological assessment made with AVPU;

Standards for Reporting Qualitative Research (SRQR)*

http://www.equator-network.org/reporting-guidelines/srqr/

Title and abstract

	Page/line no(s).
**Title** - Concise description of the nature and topic of the study Identifying the study as qualitative or indicating the approach (e.g., ethnography, grounded theory) or data collection methods (e.g., interview, focus group) is recommended	1
**Abstract** - Summary of key elements of the study using the abstract format of the intended publication; typically includes background, purpose, methods, results, and conclusions	4

Introduction

**Problem formulation** - Description and significance of the problem/phenomenon studied; review of relevant theory and empirical work; problem statement	6
**Purpose or research question** - Purpose of the study and specific objectives or questions	6

Methods

**Qualitative approach and research paradigm** - Qualitative approach (e.g., ethnography, grounded theory, case study, phenomenology, narrative research) and guiding theory if appropriate; identifying the research paradigm (e.g., postpositivist, constructivist/ interpretivist) is also recommended; rationale**	9
**Researcher characteristics and reflexivity** - Researchers' characteristics that may influence the research, including personal attributes, qualifications/experience, relationship with participants, assumptions, and/or presuppositions; potential or actual interaction between researchers' characteristics and the research questions, approach, methods, results, and/or transferability	9
**Context** - Setting/site and salient contextual factors; rationale**	7
**Sampling strategy** - How and why research participants, documents, or events were selected; criteria for deciding when no further sampling was necessary (e.g., sampling saturation); rationale**	8
**Ethical issues pertaining to human subjects** - Documentation of approval by an appropriate ethics review board and participant consent, or explanation for lack thereof; other confidentiality and data security issues	7
**Data collection methods** - Types of data collected; details of data collection procedures including (as appropriate) start and stop dates of data collection and analysis, iterative process, triangulation of sources/methods, and modification of procedures in response to evolving study findings; rationale**	8-9

For peer review only - http://bmjopen.bmj.com/site/about/guidelines.xhtml

**Data collection instruments and technologies** - Description of instruments (e.g., interview guides, questionnaires) and devices (e.g., audio recorders) used for data collection; if/how the instrument(s) changed over the course of the study	8
**Units of study** - Number and relevant characteristics of participants, documents, or events included in the study; level of participation (could be reported in results)	10
**Data processing** - Methods for processing data prior to and during analysis, including transcription, data entry, data management and security, verification of data integrity, data coding, and anonymization/de-identification of excerpts	8-9
**Data analysis** - Process by which inferences, themes, etc., were identified and developed, including the researchers involved in data analysis; usually references a specific paradigm or approach; rationale**	9-10
**Techniques to enhance trustworthiness** - Techniques to enhance trustworthiness and credibility of data analysis (e.g., member checking, audit trail, triangulation); rationale**	9-10

Results/findings

**Synthesis and interpretation** - Main findings (e.g., interpretations, inferences, and themes); might include development of a theory or model, or integration with prior research or theory	13
**Links to empirical data** - Evidence (e.g., quotes, field notes, text excerpts, photographs) to substantiate analytic findings	14-15

Discussion

**Integration with prior work, implications, transferability, and contribution(s) to the field -** Short summary of main findings; explanation of how findings and conclusions connect to, support, elaborate on, or challenge conclusions of earlier scholarship; discussion of scope of application/generalizability; identification of unique contribution(s) to scholarship in a discipline or field	15-16
**Limitations** - Trustworthiness and limitations of findings	17

Other

**Conflicts of interest** - Potential sources of influence or perceived influence on study conduct and conclusions; how these were managed	20
**Funding** - Sources of funding and other support; role of funders in data collection, interpretation, and reporting	20

*The authors created the SRQR by searching the literature to identify guidelines, reporting standards, and critical appraisal criteria for qualitative research; reviewing the reference lists of retrieved sources; and contacting experts to gain feedback. The SRQR aims to improve the transparency of all aspects of qualitative research by providing clear standards for reporting qualitative research.

For peer review only - http://bmjopen.bmj.com/site/about/guidelines.xhtml

For peer review only

**The rationale should briefly discuss the justification for choosing that theory, approach, method, or technique rather than other options available, the assumptions and limitations implicit in those choices, and how those choices influence study conclusions and transferability. As appropriate, the rationale for several items might be discussed together.

Reference:
O'Brien BC, Harris IB, Beckman TJ, Reed DA, Cook DA. **Standards for reporting qualitative research: a synthesis of recommendations.** *Academic Medicine*, Vol. 89, No. 9 / Sept 2014 DOI: 10.1097/ACM.0000000000000388

For peer review only

For peer review only - http://bmjopen.bmj.com/site/about/guidelines.xhtml
